# In Vivo and In Vitro Anti-Arthritic Effects of Cardenolide-Rich and Caffeoylquinic Acid-Rich Fractions of *Periploca forrestii*

**DOI:** 10.3390/molecules23081988

**Published:** 2018-08-09

**Authors:** Ting Liu, Xia Wang, Yan-Ling He, Yang Wang, Li Dong, Xue Ma, Lin Zheng, Chun-Hua Liu, Guang-Cheng Wang, Jiang Zheng, Yan-Yu Lan, Yong-Jun Li

**Affiliations:** 1 State Key Laboratory of Functions and Applications of Medicinal Plants, Key Laboratory of Pharmaceutics of Guizhou Province, Guizhou Medical University, Guiyang 550004, China; liuting@gmc.edu.cn (T.L.); dongliz@aliyun.com (L.D.); mailofzl@126.com (L.Z.); liu_hua139@126.com (C.-H.L.); wanggch123@163.com (G.-C.W.); zhengseattle@gmail.com (J.Z.); 2 Engineering Research Center for the Development and Applications of Ethnic Medicines and TCM (Ministry of Education), Guizhou Medical University, Guiyang 550004, China; wangx5212141@163.com (X.W.); 15285541570@163.com (Y.-L.H.); 15180803607@163.com (Y.W.); xuema0111@163.com (X.M.); 3 School of Pharmacy, Guizhou Medical University, Guiyang 550004, China

**Keywords:** rheumatoid arthritis, *Periploca forrestii*, cardenolide-rich fraction, caffeoylquinic acid-rich fraction, mitogen-activated protein kinase, nuclear factor κB

## Abstract

*Periploca forrestii* Schltr. (*P. forrestii*) is a species used in Traditional Chinese Medicine (TCM) known as “Miao medicine”, and has a long history of use in the treatment of rheumatism, rheumatoid arthritis (RA), and joint pain. The present study aimed to evaluate the anti-arthritis effects of the cardenolide-rich and caffeoylquinic acid-rich fractions (CDLFs and CQAFs) of *P. forrestii* in collagen-induced arthritic (CIA) rats, and defined the mechanisms of therapeutic action in MH7A cells treated with TNF-α. Serum rheumatoid factor (RF), TNF-α, IL-6, IL-1β, PGE_2_, NO, SOD, and MDA were determined by ELISA or other commercially assay kits. Histopathological changes in ankle joint tissues were examined. The mRNA expressions of *IL-1β*, *IL-6*, *COX-2*, and *iNOS* in MH7A cells were measured by qRT-PCR assays. In addition, the expressions of iNOS, COX-2, and p65 proteins, and the phosphorylation of IκBα, p38, ERK_1/2_, and JNK proteins in MH7A cells were analyzed by Western blot. The results showed that CDLF and CQAF could suppress the paw swelling in CIA rats at different doses (125 mg/kg, 250 mg/kg, and 500 mg/kg). Histopathological examination suggests that the CDLF and CQAF significantly relieved the damage of the structure of the ankle joint in CIA rats. In addition, serum RF, TNF-α, IL-6, IL-1β, PGE_2_, NO, and MDA were decreased, along with increased activity of serum SOD. Furthermore, CDLF and CQAF downregulated the expressions of IL-1β, IL-6, COX-2, iNOS, and p65, and inhibited the phosphorylation of IκBα, p38, ERK_1/2_, and JNK in MH7A cells treated with TNF-α. These findings demonstrated that both CDLF and CQAF exhibited anti-arthritic activity, which might be associated with their inhibitory effects on the NF-κB and MAPK signaling pathways.

## 1. Introduction

Rheumatoid arthritis (RA) is a chronic autoimmune disease that is characterized by hyperplasia of the synovial cells, chronic inflammation of the synovium, and the destruction of cartilage and bone [1]. Fibroblast-like synoviocytes (FLSs) play a key role in the pathological process of RA. Abnormal activation of FLS cells leads to the formation of pannus, the degradation of bone, and the release of a large number of inflammation cytokines in the joint, such as TNF-α, IL-6, and IL-1β [2,3]. These cytokines can cause inflammation, and thus speed up the pathological process. Among them, TNF-α has been found to be one of the most important cytokines in RA, which can lead to synovial proliferation and the high expressions of related cytokines, including IL-1β, NO, and PGE_2_ [4,5]. Such pathological processes eventually result in the destruction of joint, cartilage, and bone, and even induce deformity or disability [6]. IL-6 and IL-1 (IL-1α and IL-1β) are key cytokines in RA pathogenesis. High levels of IL-6 and IL-1 have been detected in the synovial membrane and fluid of RA joints [7]. The NF-κB and MAPK signaling pathways can be activated by TNF-α and lipopolysaccharides (LPSs), thus leading to inflammation, tissue damage, and cell proliferation [8]. Early studies have demonstrated that the expressions of signaling pathway proteins, such as p65 and the phosphorylations of IκBα, p38, ERK_1/2_, and JNK were upregulated in the human rheumatoid arthritis synovial cell line MH7A induced by TNF-α [9].

Nonsteroidal anti-inflammatory drugs, disease modifying antirheumatic drugs, adrenocorticotropic hormones, and biopharmaceuticals, are widely used to treat RA, mainly by alleviating the suffering of the patients. However, the long-term use of these drugs is costly, and may cause significant adverse reactions. Great attention has been paid to TCM for the treatment of RA, due to its low expense and lesser side effects [10,11]. *P. forrestii* belongs to the Asclepiadaceae family and is a well-known herb of TCM, which has been widely used by the “Miao” nation in China for the treatment of many diseases, such as rheumatic joint pain, soft tissue injury, and abnormal menstruation [12]. Some bioactive compounds have been found from this plant, including cardiac glycosides, oligosaccharides, coumarins, flavonoids, and triterpenoids [13,14]. It has been demonstrated that *P. forrestii* can suppress cytokine production in adjuvant-induced arthritic rats [15]. Our previous research found that the ethanol extracts of *P. forrestii* contained a large number of caffeoylquinic acids [16] and exerted an anti-inflammatory effect in LPS-induced RAW264.7 cells by inhibiting the NF-κB and MPKA pathways [17]. The extracts of *P. forrestii* stems also showed a remarkable therapeutic action in collagen-induced arthritic (CIA) rats by inhibiting the activation of Src and nuclear translocation of NF-κB [18]. However, the effective anti-arthritic fractions of *P. forrestii* ethanol extracts are still unclear, which encouraged us to explore its anti-arthritic material basis and mechanisms of therapeutic action. Our preliminary data demonstrated that the cardenolide-rich fractions (CDLFs) and caffeoylquinic acid-rich fractions (CQAFs), obtained from ethanol extracts of *P. forrestii*, could inhibit the activation of MH7A cells treated with TNF-α.

The objectives of the present study included the evaluation of the anti-arthritic effects of the CDLF and CQAF in a CIA rat model, and the investigation of its underlying anti-arthritic mechanisms in MH7A cells treated with TNF-α. 

## 2. Results

### 2.1. Effects of CDLF and CQAF on Weight, Paw Edema, and Histopathological Alterations

Compared with the non-treated control rats, the weights of non-treated control rats significantly decreased from day 15. The decrease of weight was reversed by CDLF and CQAF treatments, and there was no significant difference between the weight of the CDLF- or CQAF-treated rats and non-treated control rats (Supplementary Materials Table S1).

**Figure 1:**
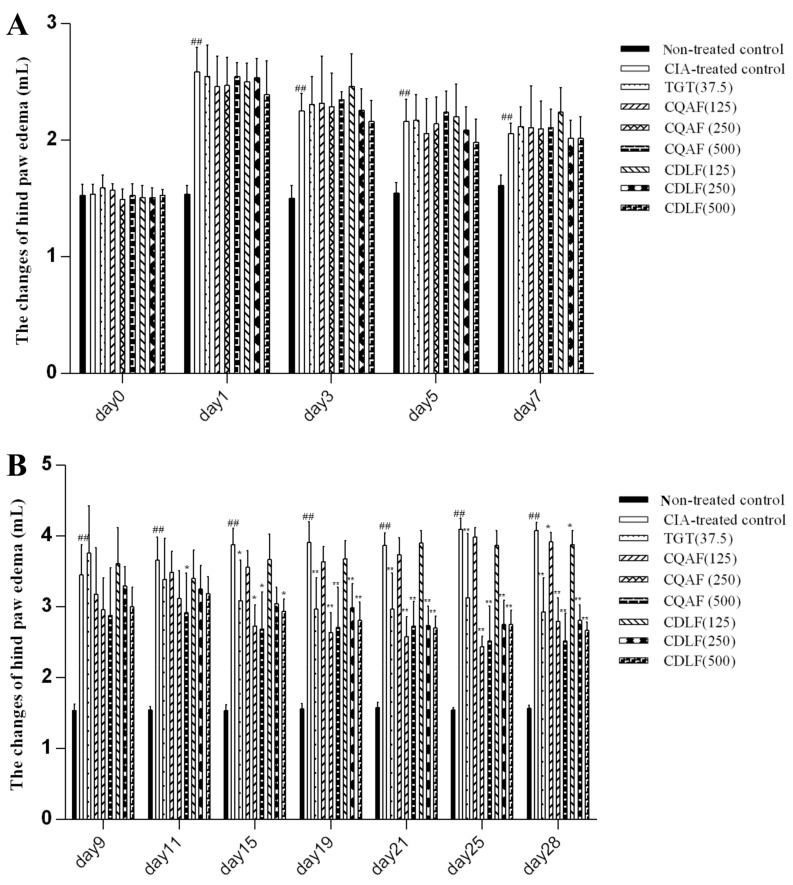
Volume of hind paw edema in rats. (**A**) days 0–7; (**B**) days 9–28. Values are represented as the mean ± SD (*n* = 8). ^##^ *p* < 0.01 when compared with the non-treated control group; * *p* < 0.05, ** *p* < 0.01 when compared with collagen-induced arthritic (CIA)-treated control group. Numeric data can be found in Supplementary Materials Table S2.

**Figure 2:**
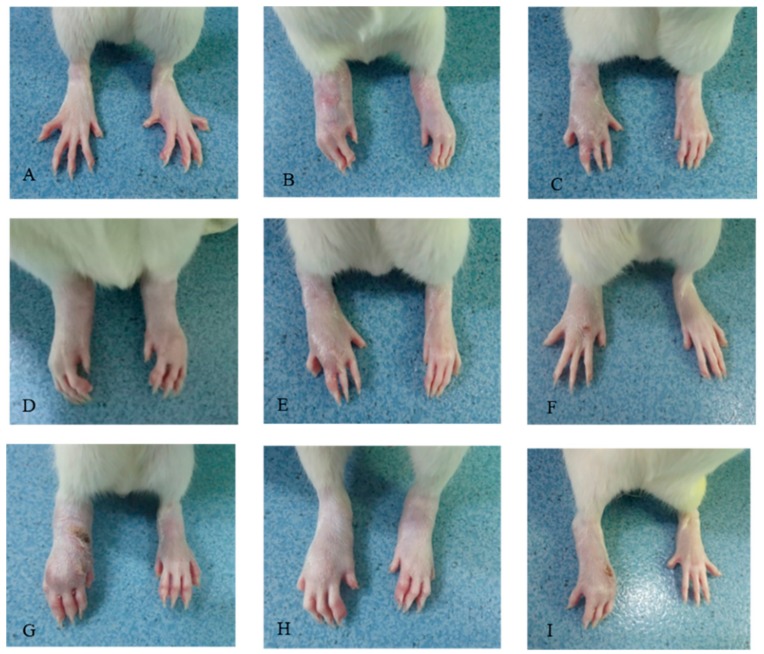
Effects of cardenolide-rich fraction (CDLF) and caffeoylquinic acid-rich fraction (CQAF) on paw edema in CIA rats. (**A**) Non-treated group; (**B**) CIA-treated control group; (**C**) tripterygium glucosides (TGT) treated (37.5 mg/kg); (**D**) CQAF treated (125 mg/kg); (**E**) CQAF treated (250 mg/kg); (**F**) CQAF treated (500 mg/kg); (**G**) CDLF treated (125 mg/kg); (**H**) CDLF treated (250 mg/kg); and (**I**) CDLF treated (500 mg/kg).

The changes of paw edema from day 0 to 28 were determined and are presented in Figure 1 and Figure 2. The right hind paws of CIA rats significantly swelled from day 1 to the end of the experiment, when compared with those of the non-treated control rats (*p* < 0.01). CDLF and CQAF significantly attenuated paw edema from day 11 (*p* < 0.05, *p* < 0.01) in a dose-dependent manner. 

**Figure 3:**
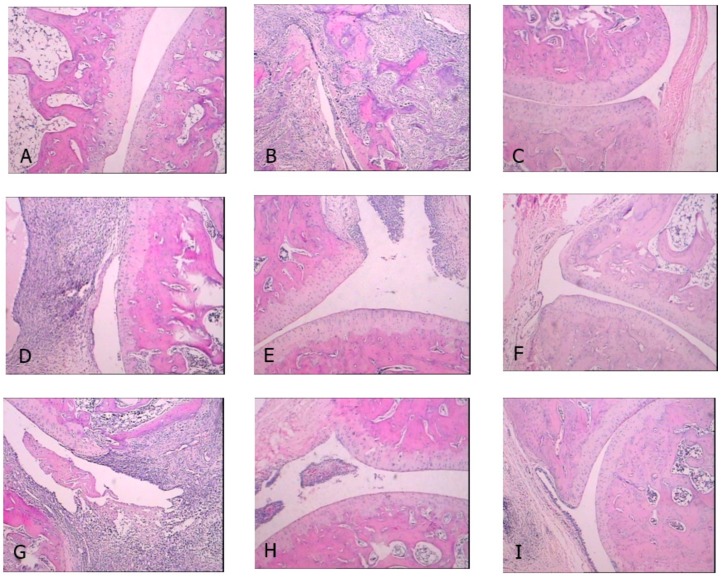
Effects of CDLF and CQAF on histopathological changes of ankle joints in CIA rats at day 28 (40×, hematoxylin and eosin (H&E) staining). (**A**) Non-treated group; (**B**) CIA-treated control group; (**C**) TGT treated (37.5 mg/kg); (**D**) CQAF treated (125 mg/kg); (**E**) CQAF treated (250 mg/kg); (**F**) CQAF treated (500 mg/kg); (**G**) CDLF treated (125 mg/kg); (**H**) CDLF treated (250 mg/kg); and (**I**) CDLF treated (500 mg/kg).

As shown in Figure 3, there was no proliferation of fibrous tissue and inflammatory infiltration in the joint structure of the non-treated control group. In the CIA-treated control group, the joint structure was severely damaged, and was accompanied with severe denaturation necrosis of chondrocyte, hyperplasia of the fibrous tissues, and massive inflammatory cell infiltration. The CDLF and CQAF treatments were found to attenuate the histopathological alterations and reduce damage of the joint structure.

### 2.2. Protective Effects of CDLF and CQAF on Inflammation

**Figure 4:**
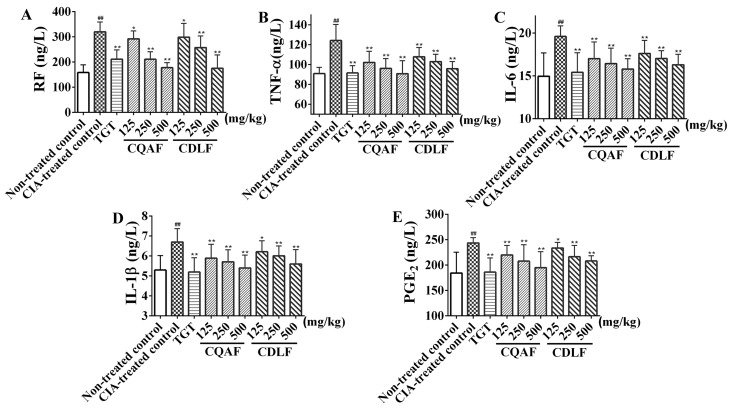
Effects of CDLF and CQAF on serum RF, TNF-α, IL-6, IL-1β, and PGE_2_ in the CIA rats. (**A**) RF; (**B**) TNF-α; (**C**) IL-6; (**D**) IL-1β; (**E**) PGE_2_. The data are presented as the mean ± SD (*n* = 8). ^##^ *p* < 0.01 when compared with the non-treated control group; * *p* < 0.05, ** *p* < 0.01 when compared with the CIA-treated control group. Numeric data can be found in Supplementary Materials Table S3.

To investigate the anti-arthritic activity of CDLF and CQAF and the mechanisms of their therapeutic action, the levels of serum RF, TNF-α, IL-6, IL-1β, and PGE_2_ were determined by ELISA assays. As shown in Figure 4, the release of RF, TNF-α, IL-6, IL-1β, and PGE_2_ in the CIA-treated control group was significantly higher than that of the non-treated control group (*p* < 0.01). Reduced production of serum RF, TNF-α, IL-6, IL-1β, and PGE_2_ in a dose-dependent manner was observed in both the CDLF and CQAF groups (*p* < 0.05, *p* < 0.01).

### 2.3. Effects of CDLF and CQAF on Serum NO, SOD, and MDA

**Figure 5:**
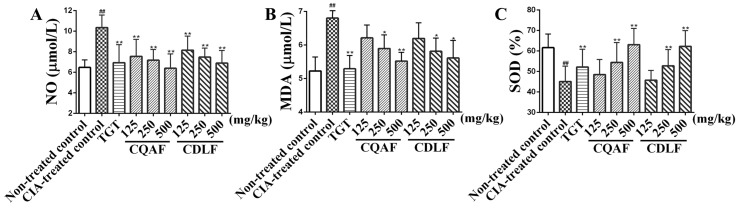
Effects of CDLF and CQAF on serum NO, MDA, and SOD in CIA rats. (**A**) NO; (**B**) MDA; (**C**) SOD. The data are presented as the mean ± SD (*n =* 8). ^##^ *p* < 0.01 when compared with non-treated control group; * *p* < 0.05, ** *p* < 0.01 when compared with CIA-treated control group. Numeric data can be found in Supplementary Materials Table S4.

The levels of NO, SOD, and MDA in sera were determined by the corresponding assay kits. As shown in Figure 5, elevated serum NO and MDA, along with decreased SOD activity, were observed in the CIA-treated control group, relative to that of the non-treated control group (*p* < 0.01). CDLF and CQAF were found to reverse the increased production of NO and MDA (*p* < 0.05, *p* < 0.01) and decreased activity of SOD (*p* < 0.01) occurring in the CIA-treated control rats. The observed anti-ROS effects appeared in a dose-dependent manner.

### 2.4. Effects of CDLF and CQAF on the Expressions of IL-1β, IL-6, iNOS, and COX-2 in MH7A Cells

**Figure 6:**
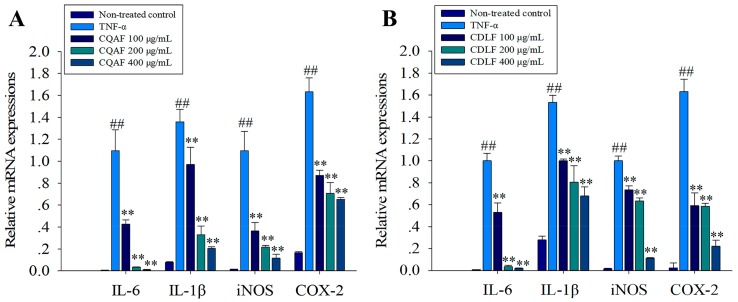
Effects of CDLF and CQAF on the mRNA expressions of *IL-1β*, *IL-6*, *iNOS*, and *COX-2* in MH7A cells. The cells were treated with CDLF or CQAF at various concentrations (100–400 μg/mL) for 24 h in the presence of TNF-α (50 ng/mL). GAPDH was used as the internal control. (**A**) CQAF treatment; (**B**) CDLF treatment. The data are presented as the mean ± SD (*n* = 3). ^##^ *p* < 0.01 when compared with the non-treated control group; ** *p* < 0.01 when compared with the TNF-α group. Numeric data can be found in Supplementary Materials Tables S5 and S6.

**Figure 7:**
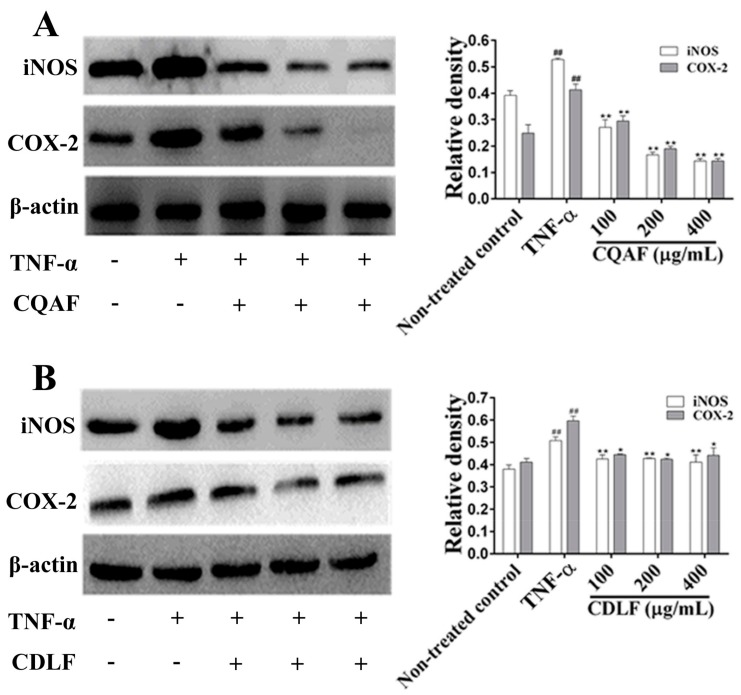
Effects of CDLF and CQAF on protein expressions of iNOS and COX-2 in MH7A cells. The cells were treated with CDLF or CQAF at various concentrations (100–400 μg/mL) for 24 h in the presence of TNF-α (50 ng/mL). β-Actin was used as the internal control. (**A**) CQAF treatment; (**B**) CDLF treatment. The data are presented as the mean ± SD (*n* = 3). ^##^ *p* < 0.01 when compared with the non-treated control group; * *p* < 0.05, ** *p* < 0.01 when compared with the TNF-α group. Original figures and numeric data can be found in Supplementary Materials Figures S1–S6 and Tables S7 and S8, respectively.

First, the mRNA expressions of *IL-1β*, *IL-6*, *iNOS*, and *COX-2* in MH7A cells were examined. Compared with the non-treated control cells, dramatic elevation in mRNA levels of *IL-1β*, *IL-6*, *iNOS*, and *COX-2* were observed in cells treated with TNF-α alone (Figure 6, *p* < 0.01). However, treatment with CDLF and CQAF significantly reduced the mRNA levels of these biomarkers (*p* < 0.01). The effects of CDLF and CQAF on the expressions of iNOS and COX-2 were further evaluated by Western blot. As expected, a significant increase in the protein expressions of iNOS and COX-2 was found in the MH7A cells treated with TNF-α (*p* < 0.01) (Figure 7), while downregulated expressions of iNOS and COX-2 (*p* < 0.05, *p* < 0.01) were observed in the cells given CDLF and CQAF. However, such effects of CDLF did not show a concentration dependency. 

### 2.5. Effects of CDLF and CQAF on the Activation of NF-κB and MAPK Pathways in MH7A Cells

**Figure 8:**
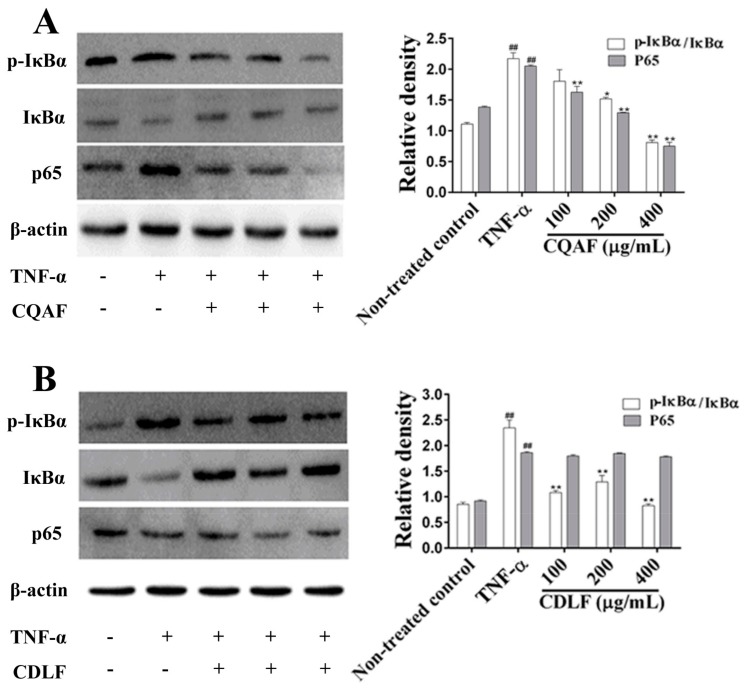
Effects of CDLF and CQAF on the expression of the p65 protein and IκB phosphorylation in MH7A cells. The cells were treated with CDLF or CQAF at various concentrations (100–400 μg/mL) for 24 h in the presence of TNF-α (50 ng/mL). β-Actin was used as the internal control. (**A**) CQAF treatment; (**B**) CDLF treatment. The data are presented as the mean ± SD (*n* = 3) when compared with the non-treated control group. ^##^ *p* < 0.01 when compared with the non-treated control group; * *p* < 0.05, ** *p* < 0.01 when compared with the TNF-α group. Original figures and numeric data can be found in Supplementary Materials Figures S7–S14 and Tables S9 and S10, respectively.

**Figure 9:**
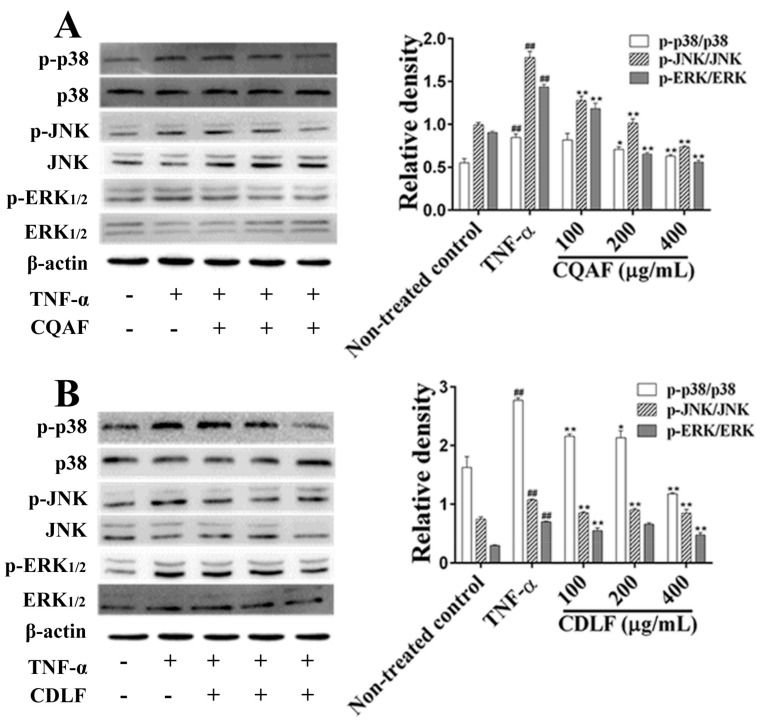
Effects of CDLF and CQAF on the protein expressions of p38, ERK, and JNK phosphorylation in MH7A cells. The cells were treated with CDLF or CQAF at various concentrations (100–400 μg/mL for 24 h in the presence of TNF-α (50 ng/mL). β-Actin was used as the internal control. (**A**) CQAF treatment; (**B**) CDLF treatment. The data are presented as the mean ± SD (*n* = 3). ^##^ *p* < 0.01 when compared with the non-treated control group, * *p* < 0.05, ** *p* < 0.01 when compared with the TNF-α group. Original figures and numeric data can be found in Supplementary Materials Figures S15–S28 and Tables S11 and S12, respectively.

To define the anti-arthritic mechanism of CDLF and CAQF, the expressions or phosphorylation of proteins involved in the NF-κB and MAPK pathways were determined by Western blot. As shown in Figure 8, the TNF-α treatment alone significantly increased the phosphorylation level of IκBα and the expression of the p65 protein (*p* < 0.01). However, co-treatment with CQAF was found to reduce the expression of the p65 protein and IκBα phosphorylation in a concentration-dependent manner (*p* < 0.05). In contrast, co-treatment with CDLF reduced IκBα phosphorylation (*p* < 0.05), but showed little effect on the expression of the p65 protein (*p* > 0.05). The phosphorylation levels of three well-established MAPK subfamilies (p38, ERK_1/2_, and JNK) were analyzed (Figure 9). Like the NF-κB pathway, the phosphorylation levels of p38, ERK, and JNK were significantly elevated in the MH7A cells treated with TNF-α (*p* < 0.01). CQAF reversed the increased phosphorylation of p38, ERK, and JNK (*p* < 0.05). CDLF exhibited the same effects as CQAF, but did not show a concentration dependency.

## 3. Discussion

*P. forrestii* has long been used to treat RA in China by the “Miao” nation. Reduction of inflammation is an important approach in RA treatment [19], and our previous study found that the ethanol extracts of *P. forrestii* exerted an anti-inflammatory effect [17]. In order to elucidate the effective anti-arthritic fraction of *P. forrestii* ethanol extracts and its molecular mechanisms of therapeutic action, the present study focused on the investigation of the anti-arthritic effects of the CDLF and CQAF obtained from ethanol extracts and their inhibitory effects on the release of inflammatory cytokines, and the activation of NF-κB and MAPK pathways.

RA is a chronic inflammatory disease characterized by pain, swelling, and joint destruction, of which severe cases can lead to deformity and even disability [11,20]. As the pathological characteristics and processes of the CIA model are very similar to clinical human rheumatoid arthritis [21,22], the CIA model is one of the models widely used for RA study and was employed to evaluate the anti-arthritic effects of CDLF and CQAF in the present study. Joint destruction, synovial hyperplasia, and inflammatory infiltration are important characteristics of the RA pathological process [23]. RF that can cause damage to synovial tissue [24,25] is an important indicator for RA. In our study, CIA rats showed severe joint damage, synovial hyperplasia, granulomatous formation, inflammatory cell infiltration, and an elevated serum level of RF, which suggests that the CIA model was successfully established. The present study also demonstrated that both CDLF and CQAF could effectively relieve paw edema, alleviate the damage of the joint structure, and lower the serum level of RA in CIA rats. Clearly, the results indicate that the CDLF and CQAF fractions had promising anti-arthritic activity.

Elevated oxidative stress factors, such as MDA and NO, are often considered as biomarkers of inflammation, along with decreased the activity of SOD [21]. A decrease in SOD activity and increases in serum MDA and NO were observed in CIA rats. CDLF and CQAF could reverse the decreased activity of SOD and increased serum MDA and NO. As NO is mainly produced by iNOS during inflammation [2], the decrease of NO production might result from the inhibitory effect of CDLF and CQAF on iNOS protein expression. The results suggest that CDLF and CQAF may reduce the oxidative damage concurrent with inflammation.

Some immune regulatory cytokines, such as TNF-α, IL-6, IL-1β, and PGE_2_, play a critical role in the pathogenesis of RA. The overexpression of TNF-α causes synovial inflammation and joint damage [26]. TNF-α also induces fibro-like synovial cells to produce a large amount of PGE_2_, as well as various cytokines, including TNF-α, IL-6, and IL-1β [27]. These inflammatory factors amplify the inflammatory response of FLS cells initially stimulated by TNF-α, which have been reported to form a vicious cycle, maintain synovial inflammation, and cause joint damage [28]. Previous study has demonstrated that inhibitors of these inflammatory factors might exert remarkable therapeutic effects in the clinical treatment of RAe [29], including *Schisandra glaucescens* [10], *Claoxylon indicum* [30], *Cinnamon cortex*, *Persica semen*, and *Natril sulfas* [31] et al. In this study, both CDLF and CQAF were found to downregulate mRNA and protein expressions of IL-6, IL-1β, and suppress the production of PGE_2_ via decreased expression of COX-2. The production of TNF-α was also slowed down by CDLF and CQAF. These results suggest anti-inflammatory effects of CDLF and CQAF, which might be involved in their anti-arthritic effects.

Although we demonstrated that the anti-arthritic effects of CDLF and CQAF might result from their anti-inflammatory activity, their underlying mechanisms need to be clarified. Our previous study [17] allowed us to pay more attention to the NF-κB and MAPK pathways. NF-κB plays a key role in the pathogenesis and progression of inflammatory reactive diseases [32]. The NF-κB protein is inactivated by IκB in the cytoplasm. When IκBα are deliberately phosphorylated, the NF-κB pathway is activated [31]. The MAPK pathway is critical to some chronic inflammatory diseases, like RA [33]. The MAPK family includes extracellular signal-regulated kinases ERK1 and ERK2, c-Jun NH_2_-terminal kinases JNK1, JNK2, and JNK3, and p38 kinases [34]. These are mainly involved in cell migration, apoptosis, differentiation, and proliferation [35]. Some plants showed anti-arthritic effects by modulating the NF-κB or MAPK pathways, such as *Glorisa superba* [4], Asarum [36], and *Bauhinia championii* [11,32]. In the present study, CQAF was found to suppress the activation of NF-κB, possibly by reducing the phosphorylation of IκBα and expression of p65. However, no such downregulated expression of p65 was observed in the MH7A cells treated with CDLF. Interestingly, treatment with CDLF did show an inhibition of IκBα phosphorylation, but the observed inhibition did not reveal concentration dependency. In addition, CDLF and CQAF remarkably inhibited the phosphorylation of p38, ERK_1/2_, and JNK. These results suggest that CDLF and CQAF might reduce inflammation in TNF-α-treated MH7A cells via downregulating NF-κB and MAPK activation, which was consistent with our previous finding [17] where ethanol extracts of *P. forrestii* exerted an anti-inflammatory effect in LPS-treated RAW264.7 cells by inhibiting the NF-κB and MAPK pathways.

It has been reported that polyphenolics such as chlorogenic acid, caffeic acid, and protocatechuic acid possess anti-inflammatory activity [37]. The treatment of caffeoylquinic acid could attenuate the severity of articular degradation in a MIA-induced OA model by downregulating catabolic activity and oxidative damage in chondrocytes [38]. Liu et al. reported that saponin from *P. forrestii* significantly ameliorated murine CFA-induced arthritis by suppressing cytokine production [15]. LC-MS results indicated the presence of pharmacologically important caffeoylquinic acid in CQAF, such as chlorogenic acid, 3,5-*O*-dicaffeoulquinic acid, and 3-*O*-caffeoylquinic acid. CDLF was also found to be rich in glycosides such as periplogenin A and periplogenin. These components might be responsible for the anti-arthritic activity of *P. forrestii*, which is consistent with the observed anti-arthritic effects of CDLF and CQAF in the present study.

## 4. Materials and Methods

### 4.1. Chemical and Reagents

Human recombinant TNF-α was purchased from PeproTech, Inc. (Rocky Hill, NJ, USA). TGT was obtained from Qianjin Xieli Pharmaceutical Co. Ltd. (Zhuzhou, China). Collagen type II from bovine nasal septum (CII) and Freund’s adjuvant complete (CFA) were purchased from Sigma-Aldrich Co. (St. Louis, MO, USA). The NO assay kit was obtained from Beyotime Biotechnology (Shanghai, China). ELISA kits for PGE_2_, IL-1β, TNF-α, and IL-6, and assay kits for SOD and MDA were supplied by Nanjing Jiancheng Biotechnology (Shanghai, China). Rabbit antibodies against β-actin, iNOS, COX-2, p65, IκB, phospho-IκB, ERK, phospho-ERK, p38, p-p38, JNK, p-JNK, and secondary antibody were acquired from Abcam (San Francisco, CA, USA). All other reagents used in the present study were of analytical grade.

### 4.2. Preparation of CDLF and CQAF

*P. forrestii* herbs (whole plant) were collected in September 2015 from the Guiyang Wan Dong Chinese herbal medicine market, and authenticated by associate professor Qing-de Long of Guizhou Medical University based on morphological and anatomical characteristics. A voucher specimen (20150901) was stored at the Key Laboratory of Pharmaceutics of Guizhou Province, Guizhou Medical University, and their images can be found in Figures S29–S31 in the Supplementary Materials. The roots and stalks of the plant were employed for extraction. The dried roots and stalks (15 kg) were extracted three times by refluxing in 70% ethanol for 1.5 h (120 L, 90 L, and 90 L of 70% ethanol, respectively). The resulting filtrates were pooled and condensed to dryness under reduced pressure, yielding 1920.5 g of crude extracts. The crude extracts were then resuspended in 12 L of water. The resultant precipitates were dried in a vacuum oven to obtain CDLF (292.5 g, yield: 1.95%). The soluble part was acidified to pH 5, loaded on D_101_ macroporous resins, washed with 80% ethanol, and further dried in a vacuum oven to obtain CQAF (641.7 g, yield: 4.3%).

UPLC-QTOF-MS analysis was applied in the chemical profiling of CDLF and CQAF in both negative and positive ion modes. The samples were separated with an ACQUITY UPLC^TM^ I-Class system (Waters, Milford, MA, USA) on an HSS T3 column (2.1 mm, 100 mm, 1.8 μm). The mobile phase consisted of water containing 0.1% formic acid (A) and acetonitrile (B). The elution gradient was as follows: 100% A (0 min), 70% A (8 min), 1% A (12 min), 100% A (15 min). The flow rate was 0.4 mL/min and the column temperature was set at 40 °C. All the mass experiments were carried out using a Xevo G2-XS QTOF system (Waters). The acquisition parameters were: source temperature, 120 °C; desolvation temperature, 450 °C; desolvation gas flow, 800 L/h; cone gas flow, 50 L/h; spray voltage, 2.0/2.0 kV; mass range recorded, *m/z* 50–1200. Compounds were characterized by comparing the precise molecular weight, fragmentation ions, and retention time with those of the published results, with the mass error of molecular ions within 5 ppm. More details of the chemical composition of CDLF and CQAF can be found in Tables S13 and S14 and Figures S32 and S33 in the Supplementary Materials.

### 4.3. Animals

Male Sprague Dawley (SD) rats (160~180 g) were provided by the Experimental Animal Center of Guizhou Medical University. Rats were maintained at a controlled temperature (22 ± 2 °C) and humidity (50 ± 5%) under a 12 h light/dark cycle, and given free access to water and food. All experiments were carried out under the approval of the Animal Ethical Committee of Guizhou Medical University (allowance number: 1403018) and conformed with the guidelines of the National Institutes of Health for the Care and Use of Animals.

### 4.4. Induction of CIA and Treatment

After adapting to the housing conditions for seven days, rats were randomly divided into nine groups (*n* = 8) and treated with TGT in the dose of 37.5 mg/kg, CDLF or CQAF in doses of 125 mg/kg, 250 mg/kg, and 500 mg/kg. A pathologic model of arthritis was established as previously described [21,39]. Briefly, bovine type II collagen (CII) was dissolved in acetic acid (0.05 M) to a concentration of 2.0 mg/mL, then emulsified with an equal volume of complete Freund’s adjuvant (CFA). The resulting CII/CFA emulsion (0.1 mL) was intradermally injected at the right hind paws of rats on day 1. After the primary injection, the rats received the same emulsion (0.1 mL) on day 7. On day 8, the rats were orally administrated with CDLF, CQAF, and TGT once daily for 20 days. The paw edema was measured with a PV 200 Volume Meter (Techman, Chengdu, China) and the body weight was recorded from days 0 to 28.

### 4.5. Histological Examination of Ankle Joints

The rats were sacrificed on day 28. The ankle joints were harvested, fixed with 10% formaldehyde solution for 24 h, decalcified in 10% EDTA for 30 days, and embedded in paraffin. Then, the paraffined tissues were cut into sections (5 μm) and subsequently stained with hematoxylin and eosin (H&E) and observed by light microscopy.

### 4.6. Measurement of Serum Levels of Cytokines

Blood samples were collected from the femoral artery of rats, and serum RF, TNF-α, IL-6, IL-1β, and PGE_2_ were measured by ELISA assay kits, according to the manufacturer’s instructions.

### 4.7. Measurement of Serum NO, SOD, and MDA

Blood samples were collected from the femoral artery of rats, and serum NO, SOD, and MDA were assessed by commercially available kits, according to the manufacturer’s instructions.

### 4.8. Cell Culture and Treatment

Human rheumatoid arthritis synovial cell line MH7A was obtained from Jennio Biotech Co., Ltd. (Guangzhou, China), and cultured in DMEM (Gibco, Waltham, MA, USA) supplemented with 10% fetal bovine serum (FBS), 100 U/mL penicillin, and 100 μg/mL streptomycin (Gibico, Waltham, MA, USA) at 37 °C and 5% CO_2_. Cells of the 5–10th passages were employed for the experiments.

The cytotoxicity of CDLF or CQAF had been evaluated by MTT assay (more details can be found in Supplementary Materials Table S15). According to the results, 24 h treatment of CDLF or CQAF showed no cytotoxicity at the concentration of 400 μg/mL, thus concentrations of 100 μg/mL, 200 μg/mL, and 400 μg/mL were employed to carry out cell experiments. MH7A cells (5 × 10^−5^ cells/well) were seeded in 6-well plates and allowed to adhere for 24 h. The cells were treated with CDLF or CQAF in doses of 100 μg/mL, 200 μg/mL, and 400 μg/mL in the presence of 50 ng/mL TNF-α for 24 h.

### 4.9. Quantitative Real-Time PCR Analysis

The total RNA of MH7A cells was isolated with the Trizol Reagent (Invitrogen, Carlsbad, CA, USA), and transcribed into cDNA using a reverse transcriptional kit (Takara, Kyoto, Japan). qRT-PCR was conducted using a SsoFast^TM^ EvaGreen Supermix PCR Kit (Bio-rad, Hercules, CA, USA) with a CFX96 Real-Time PCR system (Bio-Rad). The cycling parameters were 95 °C for 30 s, and 40 cycles of 95 °C for 3 s and 60 °C for 30 s. *GAPDH* was employed as an internal control for normalization and relative mRNA expression was quantified using the 2^−ΔΔCt^ method. The following primers were used in the study: *GAPDH* 5′-GGAGTCCACTGGGCGTCTT-3′ (forward), 5′-AGGCTGTTGTCATACTTCTCAT-3′ (reverse); *IL-1β* 5′-CCTGTCCTGCGTGAAAGA-3′ (forward), 5′-GGGAACTGGGCAGACTCAAA-3′ (reverse); *IL-6* 5′-CCTGACCCAACCACAAATGC-3′ (forward), 5′-ATCTGAGGTGCCCATGCTAC-3′ (reverse); *COX-2* 5′-GTCCCTGAGCATCTACGGTTT-3′ (forward), 5′-CAACTGCTCATCACCCCATT-3′ (reverse); *iNOS* 5′-TCCAGGA GGACATACAGCAC-3′ (forward), 5′-CGCCCTTCCGCAGTTCT-3′ (reverse).

### 4.10. Western Blot Analysis

Proteins were isolated by lysing the cells with RIPA buffer (150 mM sodium chloride, 1% NP-40, 0.5% sodium deoxycholate, 0.1% SDS, 50 mM Tris, 1% PMSF, and a cocktail inhibitor for proteases and phosphatases), and the concentration of protein was determined by the BCA assay kit (Solarbio, Beijing, China). After SDS-PAGE separation, protein was transferred to a PVDF membrane (Millipore, Burlington, MA, USA). The PVDF membrane was blocked with 0.5% BSA for 2 h, and then incubated with the primary antibody for 2 h at room temperature. After TBST wash, the PVDF membrane was incubated with the corresponding horseradish peroxidase (HRP)-conjugated secondary antibody for 2 h at room temperature. The membrane was visualized by ECL plus (Bio-rad, Hercules, CA, USA) and examined with G:BoxChemiXL1.4 (Syngene, Cambridge, UK).

### 4.11. Statistical Analysis

All data were expressed as mean ± standard deviation (SD). Significance was assessed using SPSS version 22 (IBM, Armonk, NY, USA, 2013). Significant differences were assessed using one-way analysis of variance (ANOVA) or *t* test. *p* < 0.05 was considered to be statistically significant.

## 5. Conclusions

CDLF and CQAF demonstrated anti-arthritic effects, which might result from its capability of inflammation relief by inhibiting NF-κB and MAPK activation. These results provide a strong rationale for the further testing and validation of material basis for the anti-arthritic activity of *P. forrestii*. However, the direct link between the inhibitory activity of NF-KB and MAPK and the anti-arthritic properties needs further validation.

## Supplementary Materials

The following are available online. Figures S1–S33; Tables S1–S15.

## Abbreviations

RA	rheumatoid arthritis
FLS	fibroblast-like synoviocytes
LPS	lipopolysaccharides
MH7A	human rheumatoid arthritis synovial fibroblast cell line
CII	collagen type II from bovine nasal septum
TCM	traditional Chinese medicine
CFA	Freund’s adjuvant complete
CDLF	cardenolide-rich fractions
CQAF	caffeoylquinic-acid-rich fractions
TGT	tripterygium glycosides tablet
NF-κB	nuclear factor κB
MAPK	mitogen-activated protein kinase
