# Supplementary material for: In Vivo and In Vitro Anti-Arthritic Effects of Cardenolide-Rich and Caffeoylquinic Acid-Rich Fractions of Periploca forrestii
Source: Molecules. 2018 Aug 9;23(8):1988. doi: 10.3390/molecules23081988 (PMC6222661; doi:10.3390/molecules23081988)
Supplement: Supplementary file 1 [file molecules-23-01988-s001.pdf]

Molecules

In Vivo and In Vitro Anti-Arthritic Effects of Cardenolide-Rich and Caffeoylquinic Acid-Rich Fractions of *Periploca Forrestii*

Ting Liu[1†], Xia Wang[2, 3†], Yan-Ling He[2, 3], Yang Wang[2, 3], Li Dong[1], Xue Ma[2], Lin Zheng[1], Chun-Hua Liu[1], Guang-Cheng Wang[1], Jiang Zheng[1], Yan-Yu Lan[2*], Yong-Jun Li[2*]

[1] State Key Laboratory of Functions and Applications of Medicinal Plants, Key Laboratory of Pharmaceutics of Guizhou Province, Guizhou Medical University, Guiyang, 550004, China; t-liu@163.com (T.L.); dongliz@aliyun.com (D.L.); mailofzl@126.com (L.Z.); liu_hua139@126.com (C-H.L.); wanggch123@163.com (G-C.W.); zhengseattle@gmail.com (J.Z.)

[2] Engineering Research Center for the Development and Applications of Ethnic Medicines and TCM (Ministry of Education), Guizhou Medical University, Guiyang, 550004, China; wangx5212141@163.com (X.W.); 15285541570@163.com (Y-L.H.); 15180803607@163.com (Y.W.); xuema0111@163.com (X.M.)

[3] School of Pharmacy, Guizhou Medical University, Guiyang, 550004, China

* Correspondence: yanyu626@126.com (Y-Y.L.); liyongjun026@126.com(Y-J.L.); Tel.: +86-851-84123002 (Y-Y.L.); +86-851-8698468 (Y-J.L.)

† These authors contributed equally to this work.

Table 1-a Changes of the weight of rats from day0 to 11 ($\bar{x} \pm s$，$n = 8$)

Groups	day 0	day 1	day 3	day 5	day 7	day 9	day 11
non-treated control	138.16±8.71	163.02±9.64	164.05±9.64	165.50±9.32	184.46±10.16	175.20±9.50	182.38±6.66
CIA-treated control	135.91±8.09	159.90±12.93	159.9±12.93	167.30±7.52	184.97±12.40	172.65±14.37	174.16±9.80#
TGT (37.5 mg/kg)	137.22±9.15	165.40±12.07	165.40±12.07	163.49±12.18	184.49±15.31	174.67±13.55	178.38±9.30
CQAF (125 mg/kg)	134.17±7.37	159.90±13.60	160.90±13.20	164.50±12.90	187.60±15.30	170.85±13.26	176.05±8.34
CQAF (250 mg/kg)	135.71±6.53	157.50±10.80	161.00±9.80	161.90±10.10	182.60±10.90	169.92±8.75	177.73±4.93
CQAF (500 mg/kg)	136.97±8.27	156.00±18.70	159.30±19.60	165.50±19.10	179.80±24.40	173.37±9.76	179.52±6.48
CDLF (125 mg/kg)	135.17±8.37	161.21±8.22	161.21±8.22	167.49±9.11	185.73±9.26	169.85±16.26	174.05±8.44
CDLF (250 mg/kg)	134.71±6.93	157.43±6.06	157.43±6.06	162.29±6.68	181.67±8.44	169.72±8.85	174.73±3.93
CDLF (500 mg/kg)	135.97±8.07	156.90±11.51	156.90±11.51	163.98±11.30	183.84±14.72	173.37±8.76	180.52±4.48

Table 1-b Changes of the weight of rats from day0 to 11 ($\bar{x} \pm s$, $n = 8$)

Groups	Groups (mg/kg)	day 15	day 19	day 21	day 25	day 28
non-treated control	non-treated control	205.53±8.09	235.25±9.08	240.62±10.88	251.89±12.07	263.63±10.63
CIA-treated control	CIA-treated control	196.47±11.05$^{\#}$	217.43±9.99$^{\#\#}$	216.21±14.26$^{\#\#}$	220.82±21.53$^{\#\#}$	211.04±35.17$^{\#\#}$
TGT (37.5 mg/kg)	TGT (37.5)	201.49±13.90	231.64±14.98*	230.11±17.42	242.66±18.84*	247.29±21.10*
CQAF (125 mg/kg)	CQAF (125)	197.23±12.88	220.48±18.43	215.87±10.24	228.54±12.58	230.10±16.12*
CQAF (250 mg/kg)	CQAF (250)	200.90±6.39	226.47±7.47	229.76±7.69	235.68±15.72*	250.20±6.67**
CQAF (500 mg/kg)	CQAF (500)	203.57±5.84	228.85±9.82*	233.28±12.59*	243.61±15.61**	258.26±16.02**
CDLF (125 mg/kg)	CDLF (125)	193.23±13.88	222.48±19.43	216.47±13.24	230.24±22.58	233.10±18.22*
CDLF (250 mg/kg)	CDLF (250)	199.90±5.39	228.57±8.47*	230.79±7.69*	239.99±20.68*	255.20±6.97**
CDLF (500 mg/kg)	CDLF (500)	204.27±5.94	231.95±12.82*	237.27±15.89**	248.91±15.21**	261.29±26.02**

Values are represented as the mean ± SD (n=8). $^{\#}P$<0.05, $^{\#\#}$ P<0.01 when compared with the non-treated control group; $^{*}P$<0.05, $^{**}P$<0.01 when compared with the CIA-treated control group.

Table 2. Effects of CDLF and CQAF on hind paw edema in arthritic rats induced by collagen

Groups (mg/kg)	The changes of hind paw edema (mL)											
	day 0	day 1	day 3	day 5	day 7	day 9	day 11	day 15	day 19	day 21	day 25	day 28
non-treated control	1.53±0.09	1.54±0.07	1.50±0.11	1.55±0.09	1.61±0.09	1.54±0.09	1.55±0.05	1.54±0.08	1.56±0.08	1.58±0.07	1.55±0.03	1.57±0.04
CIA-treated control	1.54±0.08	2.59±0.21$^{\#\#}$	2.25±0.15$^{\#\#}$	2.16±0.19$^{\#\#}$	2.06±0.09$^{\#\#}$	3.45±0.43$^{\#\#}$	3.66±0.33$^{\#\#}$	3.88±0.23$^{\#\#}$	3.91±0.29$^{\#\#}$	3.87±0.18$^{\#\#}$	4.10±0.15$^{\#\#}$	4.08±0.12$^{\#\#}$
TGT (37.5)	1.59±0.11	2.55±0.27	2.31±0.24	2.17±0.22	2.12±0.17	3.76±0.67	3.39±0.58	3.09±0.57*	2.97±0.44**	2.97±0.51**	3.13±0.91**	2.93±0.48**
CQAF (125)	1.57±0.06	2.46±0.26	2.32±0.40	2.06±0.30	2.11±0.36	3.18±0.66	3.49±0.30	3.56±0.24	3.64±0.21	3.74±0.24	3.99±0.13	3.92±0.13*
CQAF (250)	1.49±0.09	2.47±0.24	2.29±0.29	2.14±0.23	2.10±0.24	2.96±0.45	3.12±0.39	2.73±0.30*	2.64±0.28**	2.58±0.28**	2.44±0.15**	2.80±0.33**
CQAF (500)	1.53±0.10	2.55±0.12	2.35±0.07	2.24±0.18	2.11±0.16	2.88±0.67	2.92±0.56*	2.69±0.52*	2.71±0.57**	2.73±0.35**	2.52±0.49**	2.52±0.39**
CDLF (125)	1.51±0.10	2.50±0.16	2.46±0.28	2.20±0.28	2.24±0.21	3.61±0.51	3.40±0.40	3.67±0.36	3.68±0.26	3.90±0.18	3.87±0.21	3.88±0.20*
CDLF (250)	1.51±0.08	2.54±0.16	2.26±0.18	2.09±0.20	2.02±0.15	3.30±0.27	3.25±0.34	3.05±0.23	2.99±0.34**	2.74±0.27**	2.75±0.35**	2.81±0.22**
CDLF (500)	1.53±0.05	2.39±0.29	2.16±0.18	1.98±0.20	2.02±0.18	3.00±0.28	3.19±0.24	2.94±0.17*	2.81±0.26**	2.70±0.17**	2.75±0.20**	2.67±0.12**

Values are represented as the mean ± SD (n=8). $^{\#\#}P$<0.01 when compared with the non-treated control group; $^{*}P$<0.05, $^{**}P$<0.01 when compared with the CIA-treated control group.

Table 3. Effects of CDLF and CQAF on serum RF, TNF-*α*, IL-6, IL-1β, and PGE_2 in the CIA rats (*n* = 8)

Group		RF (ng/L)	TNF-*α* (ng/L)	IL-6 (ng/L)	IL-1β (ng/L)	PGE_2 (ng/L)
Non-treated control	-	158.405±39.553	90.953±6.256	14.962±2.726	5.298±0.719	184.489±40.919
CIA-treated control	-	320.242±38.792$^{\#\#}$	124.253±16.238$^{\#\#}$	19.619±1.224$^{\#\#}$	6.700±0.668$^{\#\#}$	243.784±10.426$^{\#\#}$
TGT	37.5 mg/kg	211.564±36.506**	91.614±7.383**	15.425±2.278**	5.199±0.718**	186.212±28.079**
	125 mg/kg	292.285±31.388*	102.201±11.269**	17.029±1.934**	5.894±0.696**	220.118±18.604**
CQAF	250 mg/kg	211.090±29.984**	96.121±9.893**	16.443±1.808**	5.712±0.597**	207.881±32.322**
	500 mg/kg	177.832±20.065**	90.916±12.997**	15.807±1.199**	5.401±0.643**	195.132±31.437**
	125 mg/kg	298.744±54.909*	107.901±9.421**	17.629±1.513**	6.214±0.545**	233.718±11.111*
CDLF	250 mg/kg	257.517±45.910**	103.021±7.383**	17.043±0.903**	6.012±0.492**	216.581±22.107**
	500 mg/kg	175.539±52.484**	95.916±7.128**	16.307±1.221**	5.601±0.719**	208.332±10.164**

The data are presented as the mean ± SD. $^{\#\#}P < 0.01$ when compared with the non-treated control group; $^{*}P < 0.05$, $^{**}P < 0.01$ when compared with the CIA-treated control group.

Table 4. Effects of CDLF and CQAF on serum NO, MDA, and SOD in CIA rats (*n* = 8)

Group		NO (μmol/L)	MDA (μmol/L)	SOD (%)
Non-treated control	-	6.479±0.731	5.227±0.413	61.658±6.626
CIA-treated control	-	10.350±1.219$^{\#\#}$	6.805±0.390$^{\#\#}$	45.092±7.565$^{\#\#}$
TGT	37.5 mg/kg	6.938±1.757**	5.293±0.394**	52.179±8.663**
	125 mg/kg	7.553±1.660**	6.214±0.384	48.534±7.314
CQAF	250 mg/kg	7.176±1.052**	5.895±0.407*	54.448±9.716**
	500 mg/kg	6.396±1.392**	5.522±0.260**	63.097±7.948**
	125 mg/kg	8.153±1.369**	6.195±0.467	45.784±4.787
CDLF	250 mg/kg	7.476±0.875**	5.814±0.395*	52.782±7.949**
	500 mg/kg	6.896±1.223**	5.615±0.522*	62.263±7.666**

The data are presented as the mean ± SD. $^{\#\#}P<0.01$ when compared with the non-treated control group; $^{*}P<0.05$, $^{**}P<0.01$ when compared with the CIA-treated control group.

Table 5. Effects of CQAF on the mRNA expressions of IL-1β, IL-6, iNOS, and COX-2 in MH7A cells

Group		Relative mRNA expression			
		IL-6	IL-1β	iNOS	COX-2
Non-treated control	-	0.006±0.002	0.078±0.005	0.016±0.001	0.167±0.008
TNF-*α*	50 ng/mL	1.096±0.190$^{\#\#}$	1.359±0.113$^{\#\#}$	1.096±0.175$^{\#\#}$	1.631±0.127$^{\#\#}$
	100 μg/mL	0.427±0.038**	0.970±0.157**	0.363±0.079**	0.870±0.046**
CQAF	200 μg/mL	0.034±0.003**	0.330±0.078**	0.216±0.014**	0.706±0.099**
	400 μg/mL	0.011±0.001**	0.204±0.017**	0.116±0.034**	0.653±0.016**

The data are presented as the mean ± SD (*n* = 3). $^{\#\#}P < 0.01$ when compared with the non-treated control group; $^{**}P < 0.01$ when compared with the TNF-*α* group.

Table 6. Effects of CDLF on the mRNA expressions of IL-1β, IL-6, iNOS, and COX-2 in MH7A cells

Group		Relative mRNA expressions			
		IL-6	IL-1β	iNOS	COX-2
Non-treated control	-	0.007±0.002	0.279±0.034	0.015±0.003	0.023±0.031
TNF-α	50 ng/mL	1.000±0.069##	1.532±0.065##	1.000±0.043##	1.631±0.114##
	100 μg/mL	0.531±0.085**	1.000±0.016**	0.735±0.036**	0.591±0.117**
CDLF	200 μg/mL	0.034±0.015**	0.806±0.149**	0.633±0.028**	0.585±0.029**
	400 μg/mL	0.019±0.003**	0.679±0.082**	0.112±0.005**	0.221±0.054**

The data are presented as the mean ± SD (n = 3). ##$P < 0.01$ when compared with the non-treated control group; **$P < 0.01$ when compared with the TNF-α group.

Table 7. Effects of CQAF on protein expressions of iNOS and COX-2 in MH7A cells (n = 3)

Group		Relative density	
		iNOS	COX-2
Non-treated control	-	0.392±0.018	0.249±0.031
TNF-α	50 ng/mL	0.527±0.005##	0.412±0.022##
	100 μg/mL	0.271±0.029**	0.294±0.020**
CQAF	200 μg/mL	0.167±0.010**	0.189±0.009**
	400 μg/mL	0.143±0.009**	0.143±0.009**

The data are presented as the mean ± SD. ##$P < 0.01$ when compared with the non-treated control group; **$P < 0.01$ when compared with the TNF-α group.

Table 8. Effects of CDLF on protein expressions of iNOS and COX-2 in MH7A cells (n = 3)

Group		Relative density	
		iNOS	COX-2
Non-treated control	-	0.380±0.019	0.411±0.017
TNF-α	50 ng/mL	0.508±0.016##	0.596±0.021##
	100 μg/mL	0.425±0.019**	0.445±0.003*
CDLF	200 μg/mL	0.428±0.002**	0.424±0.005*
	400 μg/mL	0.411±0.032**	0.441±0.034*

The data are presented as the mean ± SD. ##$P < 0.01$ when compared with the non-treated control group; *$P < 0.05$, **$P < 0.01$ when compared with the TNF-α group.

Table 9. Effects of CAQF on the expression of the p65 protein and IκB phosphorylation (n = 3)

Group		Relative density	
		p-IkBα/IkBα	P65
Non-treated control	-	1.109±0.029	1.387±0.015
TNF-α	50 ng/mL	2.175±0.096##	2.050±0.017##
	100 μg/mL	1.808±0.185**	1.628±0.095*
CAQF	200 μg/mL	1.518±0.026**	1.296±0.006*
	400 μg/mL	0.811±0.041**	0.752±0.061*

The data are presented as the mean ± SD (n = 3). ##$P < 0.01$ when compared with the non-treated control group; *$P < 0.05$, **$P < 0.01$ when compared with the TNF-α group.

Table 10. Effects of CDLF on the expression of the p65 protein and IκB phosphorylation (*n* = 3)

Group		Relative density	
		p-IkBα/IkBα	P65
Non-treated control	-	0.855±0.037	0.916±0.022
TNF-α	50 ng/mL	2.346±0.154##	1.864±0.019##
	100 μg/mL	1.085±0.039**	1.799±0.025
CDLF	200 μg/mL	1.293±0.126**	1.847±0.012
	400 μg/mL	0.828±0.036**	1.783±0.016

The data are presented as the mean ± SD (*n* = 3). ##*P* < 0.01 when compared with the non-treated control group; ***P* < 0.01 when compared with the TNF-α group.

Table 11. Effects of CQAF on the protein expressions of p38, ERK, and JNK phosphorylation in MH7A cells (*n* = 3)

Group		p-p38/p38 (relative density)	p-JNK/JNK (relative density)	p-ERK/ERK (relative density)
Non-treated control	-	0.551±0.049	0.996±0.024	0.905±0.017
TNF-α	50 ng/mL	0.847±0.043##	1.777±0.073##	1.436±0.030##
	100 μg/mL	0.819±0.077	1.277±0.051**	1.183±0.065**
CAQF	200 μg/mL	0.708±0.030*	1.015±0.051**	0.654±0.018**
	400 μg/mL	0.631±0.015**	0.736±0.012**	0.560±0.022**

The data are presented as the mean ± SD (*n* = 3). ##*P* < 0.01 when compared with the non-treated control group; **P* < 0.05, ***P* < 0.01 when compared with the TNF-α group.

Table 12. Effects of CDLF on the protein expressions of p38, ERK, and JNK phosphorylation in MH7A cells (*n* = 3)

Group		p-p38/p38 (relative density)	p-JNK/JNK (relative density)	p-ERK/ERK (relative density)
Non-treated control	-	1.628±0.183	0.746±0.040	0.296±0.012
TNF-α	50 ng/mL	2.775±0.040##	1.071±0.017##	0.704±0.011##
	100 μg/mL	2.155±0.044**	0.854±0.021**	0.551±0.048**
CDLF	200 μg/mL	2.134±0.117*	0.901±0.025**	0.662±0.025
	400 μg/mL	1.178±0.014**	0.848±0.063**	0.477±0.039**

The data are presented as the mean ± SD (*n* = 3). ##*P* < 0.01 when compared with Non-treated control group; **P* < 0.05, ***P* < 0.01 when compared with the TNF-α group.

Table 13. Chemical composition of CDLF

chemical formula	RT (min)	m/z （ESI-)	mass error (ppm)	response (ESI-)	adduct	m/z （ESI+)	mass error (ppm)	response (ESI+)	adduct
$C_{40}H_{66}O_{17}$	9.18	863.4311	3.3	626912	+HCOO	841.4202	1.1	116006	+Na
$C_{36}H_{56}O_{13}$	9.29	741.3707	0.6	159633	+HCOO, +Cl	719.3608	-0.7	12296	+Na, +K
$C_{23}H_{34}O_{6}$	9.43	451.2333	-0.9	201326	+HCOO, -H	ND	ND	ND	ND
$C_{23}H_{32}O_{5}$	9.63	433.223	-0.5	76661	+HCOO, -H, +Cl	389.2316	-1.6	125074	+H, +Na, +K
$C_{40}H_{66}O_{16}$	9.67	847.4353	2.4	346887	+HCOO	ND	ND	ND	ND
$C_{23}H_{34}O_{5}$	9.68	435.2386	-0.5	850781	+HCOO, -H, +Cl	391.2471	-2	397796	+H, +Na, +K
$C_{18}H_{34}O_{5}$	10.04	329.2331	-0.8	544269	-H	ND	ND	ND	ND
$C_{27}H_{44}O_{6}$	10.43	509.3119	-0.2	128993	+HCOO	487.3029	-0.2	19972	+Na, +K
$C_{34}H_{52}O_{9}$	11.03	649.3606	2	354449	+HCOO, +Cl	627.3508	0.7	91194	+Na, +K

Table 14. Chemical composition of CQAF

Compound	chemical formula	RT (min)	m/z (ESI-)	mass error (ppm)	response (ESI-)	adduct	m/z (ESI+)	mass error (ppm)	response (ESI+)	adduct
Caffeoylquinic acid /isomer	$C_{16}H_{18}O_9$	3.64	353.0875	-0.8	105705	-H	355.1014	-2.6	5575	+H,+Na
Caffeoylquinic acid /isomer	$C_{16}H_{18}O_9$	4.41	353.0876	-0.7	283059	-H	355.1017	-1.8	54538	+H,+Na,+K
Caffeoylquinic acid /isomer	$C_{16}H_{18}O_9$	4.56	353.0873	-1.4	223765	-H	355.1014	-2.6	11899	+H,+Na
3-o-caffeoyl-4-o-sinapoylquinic acid	$C_{27}H_{28}O_{13}$	5.85	559.1457	0	155297	-H	561.1591	-2.1	15928	+H
Caffeoylquinic acid ethyl ester /isomer	$C_{18}H_{22}O_9$	6.13	381.1187	-1	213130	-H	383.1327	-2.4	23139	+H
Caffeoylquinic acid ethyl ester /isomer	$C_{18}H_{22}O_9$	6.75	381.1188	-0.9	324074	-H	383.1331	-1.6	35625	+H
Caffeoylquinic acid ethyl ester /isomer	$C_{18}H_{22}O_9$	7.03	381.119	-0.3	553767	-H	383.1329	-2.1	96059	+H

Table 15. Effects of CDLF or CQAF on the viability of MH7A cells after 24 h treatment (n = 6)

Group		Cell viability (%)
Non-treated control	-	100.00±2.65
CQAF	100 μg/mL	101.23±3.15
	200 μg/mL	100.36±2.83
	300 μg/mL	99.89±3.74
	400 μg/mL	99.24±3.39
	500 μg/mL	95.32±0.77*
	600 μg/mL	93.64±0.89**
CDLF	100 μg/mL	100.09±0.68
	200 μg/mL	100.87±1.46
	300 μg/mL	101.29±2.16
	400 μg/mL	99.89±0.96
	500 μg/mL	94.07±1.32**
	600 μg/mL	92.03±1.62**

MH7A cells (1 × 10^{-4} cells/well) were seeded in 96-well plates and allowed to adhere for 24 h. The cells were then treated with CDLF or CQAF in doses of 100 μg/mL, 200 μg/mL, 300 μg/mL, 400 μg/mL, 500 μg/mL and 600 μg/mL for 24 h. After treatment, cells were incubated with 10 μL of MTT (Promega, USA) for 2 h, and then the absorbance at 490 nm was measured with a model 680 microplate reader (Bio-rad, USA). The viability was calculated with the following fomula.

Cell viability (%) = (A_{sample} – A_{blank}）/（$A_{control}$- A_{blank}）×100%。

A_{sample}: absorbance of CDLF or CQAF group at 490 nm

$A_{control}$: absorbance of the none-treated control group at 490 nm

$A_{control}$: absorbance of the cultrue medium at 490 nm

The data are presented as the mean ± SD. $^{*}P < 0.05$, $^{**}P < 0.01$ when compared with the none-treated control group.

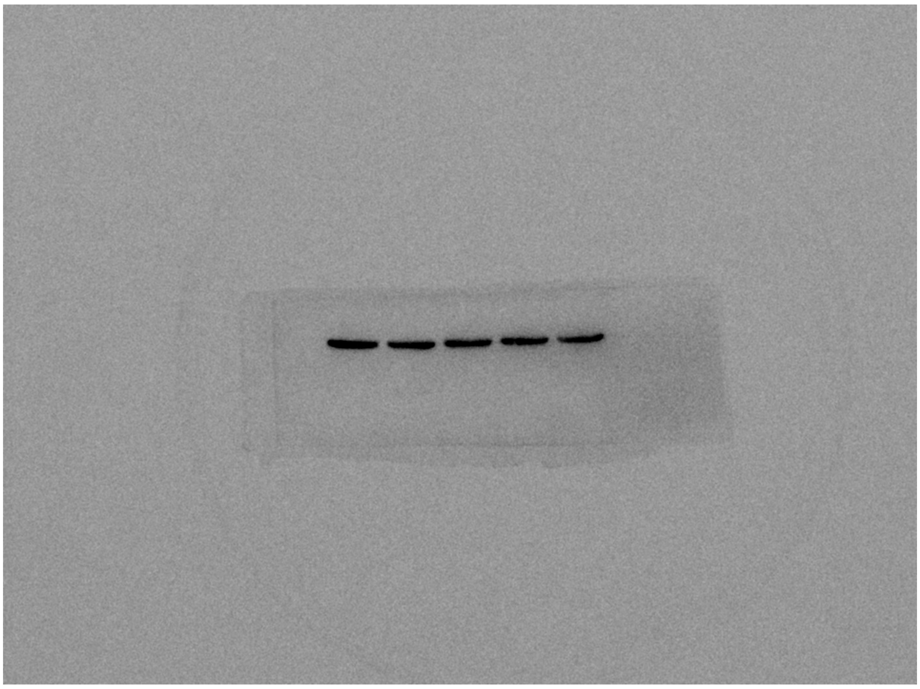

Figure 1. Original western-blot figure of Figure 7 in manuscript. CDFL, β-actin. From left to right: non-treated control group, TNF-α group, CDFL (100 μg/mL) group, CDFL (200 μg/mL) group, CDFL (400 μg/mL) group.

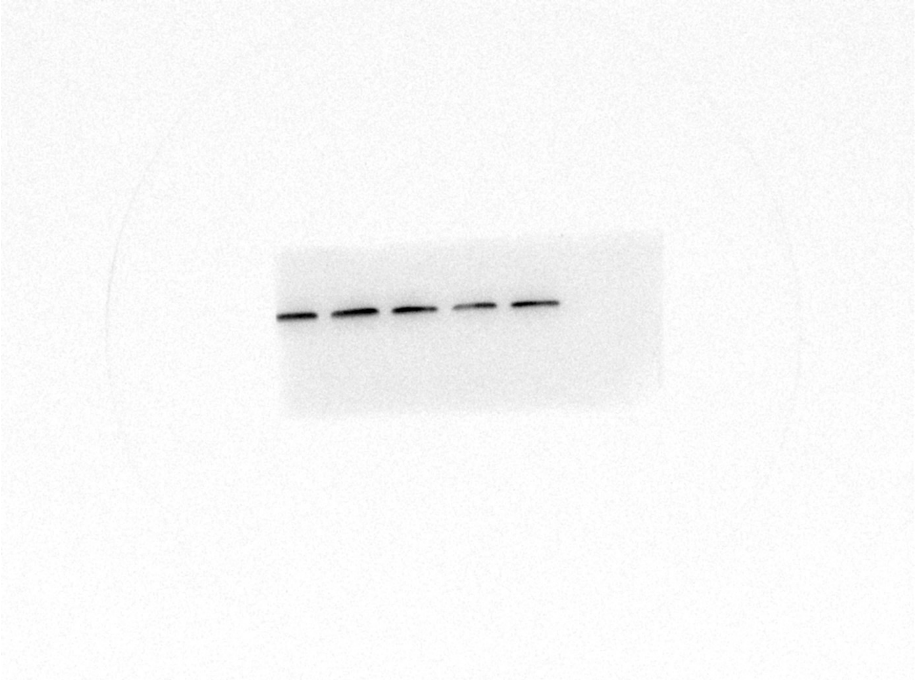

Figure 2. Original western-blot figure of Figure 7 in manuscript. CDFL, COX-2. From left to right: non-treated control group, TNF-α group, CDFL (100 μg/mL) group, CDFL (200 μg/mL) group, CDFL (400 μg/mL) group.

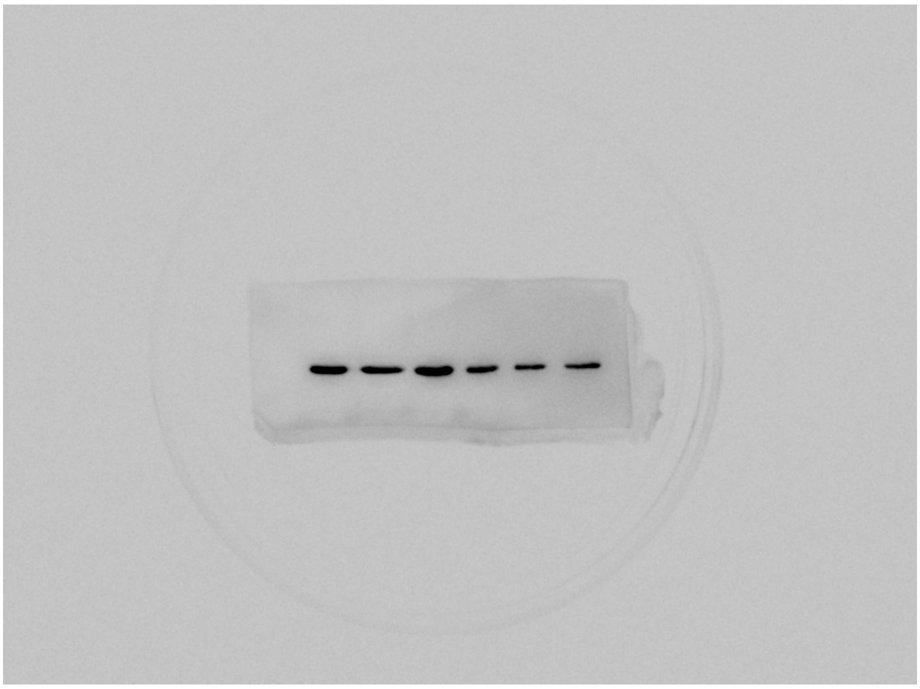

Figure 3. Original western-blot figure of Figure 7 in manuscript. CDFL, iNOS. From left to right: quality control sample, non-treated control group, TNF-α group, CDFL (100 μg/mL) group, CDFL (200 μg/mL) group, CDFL (400 μg/mL) group.

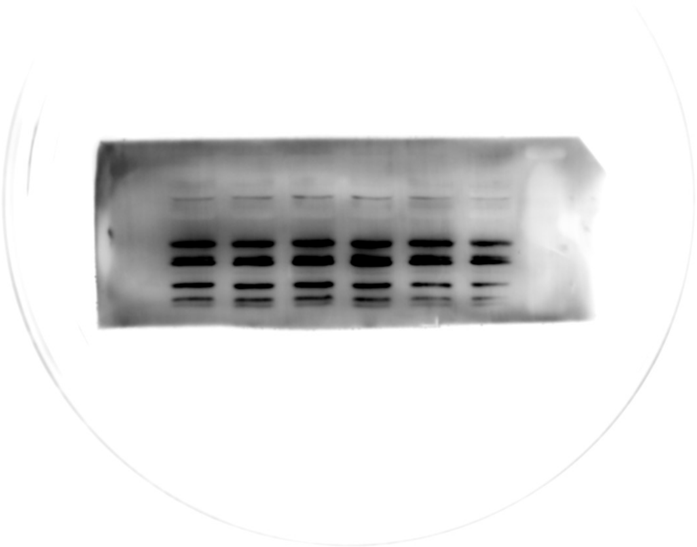

Figure.4 Original western-blot figure of Figure 7 in manuscript. CQAF, β-actin. From left to right: non-treated control group, TNF-α group, CQAF (100 μg/mL) group, CQAF (200 μg/mL) group, CQAF (400 μg/mL) group, quality control sample.

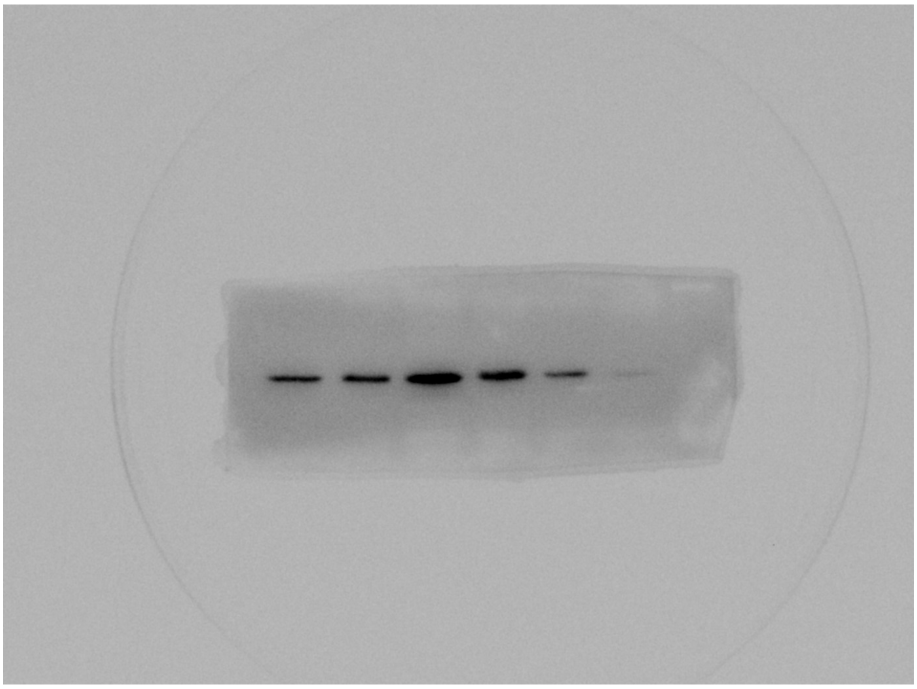

Figure 5. Original western-blot figure of Figure 7 in manuscript. CQAF, COX-2. From left to right: quality control sample, non-treated control group, TNF-α group, CQAF (100 μg/mL), CQAF (200 μg/mL), CQAF (400 μg/mL).

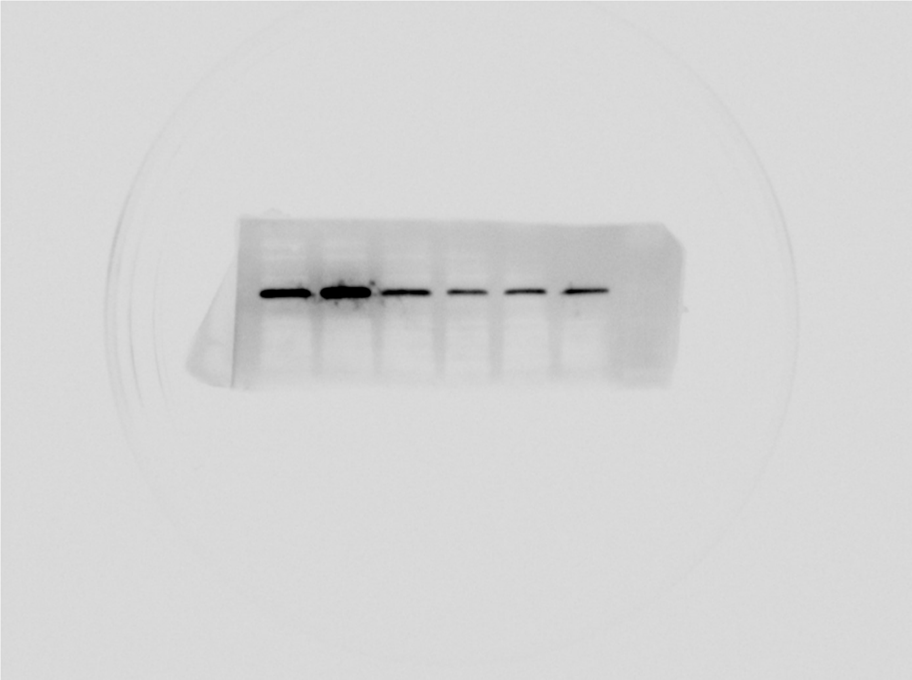

Figure 6. Original western-blot figure of Figure 7 in manuscript. CQAF, iNOS. From left to right: non-treated control group, TNF-α group, CQAF (100 μg/mL), CQAF (200 μg/mL), CQAF (400 μg/mL), quality control sample.

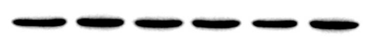

Figure 7. Original western-blot figure of Figure 8 in manuscript. CDFL, β-actin. From left to right: non-treated control group, TNF-α group, CDFL (100 μg/mL) group, CDFL (200 μg/mL) group, CDFL (400 μg/mL) group, quality control sample.

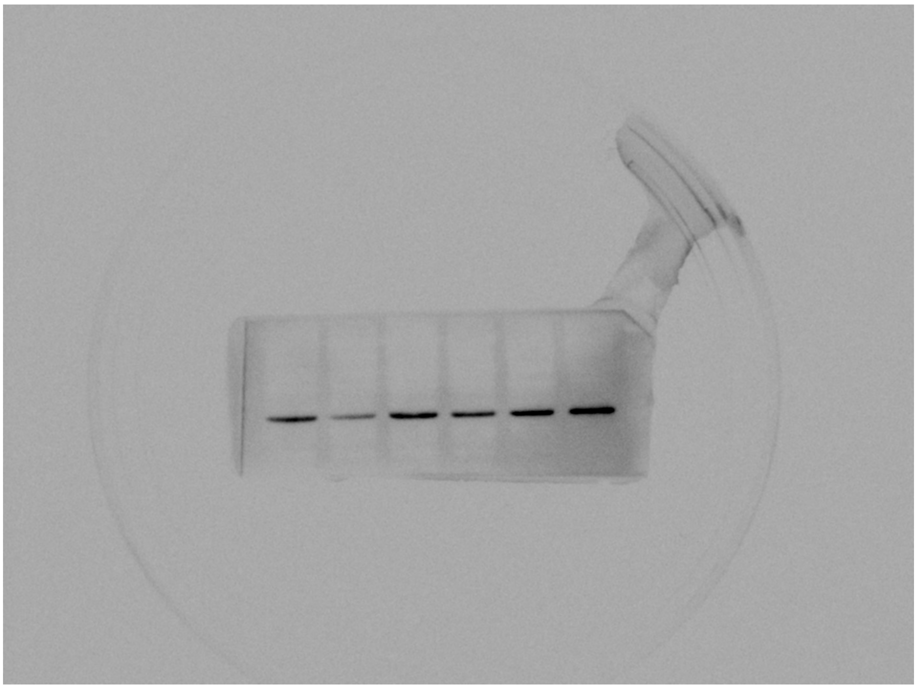

Figure 8. Original western-blot figure of Figure 8 in manuscript. CDFL, IκBα. From left to right: non-treated control group, TNF-α group, CDFL (100 μg/mL) group, CDFL (200 μg/mL) group, CDFL (400 μg/mL) group, quality control sample.

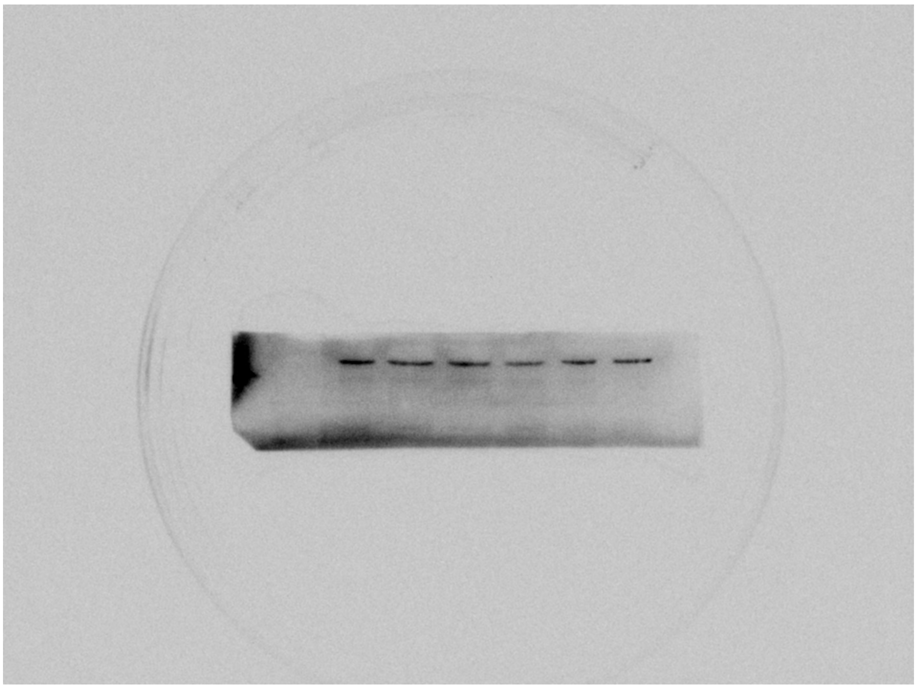

Figure 9. Original western-blot figure of Figure 8 in manuscript. CDFL, p65. From left to right: non-treated control group, TNF-α group, CDFL (100 μg/mL) group, CDFL (200 μg/mL) group, CDFL (400 μg/mL) group, quality control sample.

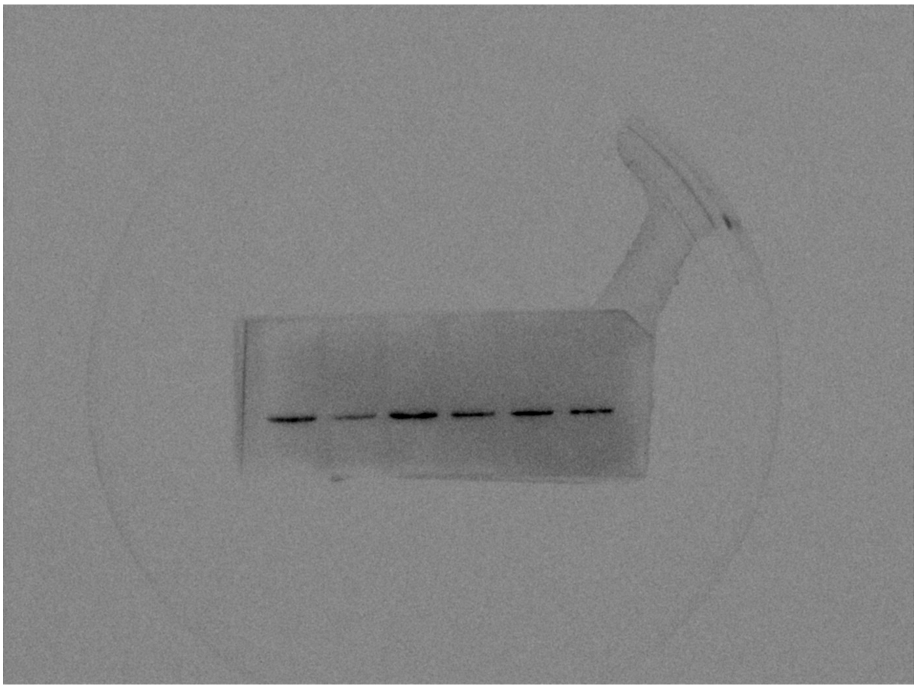

Figure 10. Original western-blot figure of Figure 8 in manuscript. CDFL, p-IκBα. From left to right: quality control sample, non-treated control group, TNF-α group, CDFL (100 μg/mL) group, CDFL (200 μg/mL) group, CDFL (400 μg/mL) group.

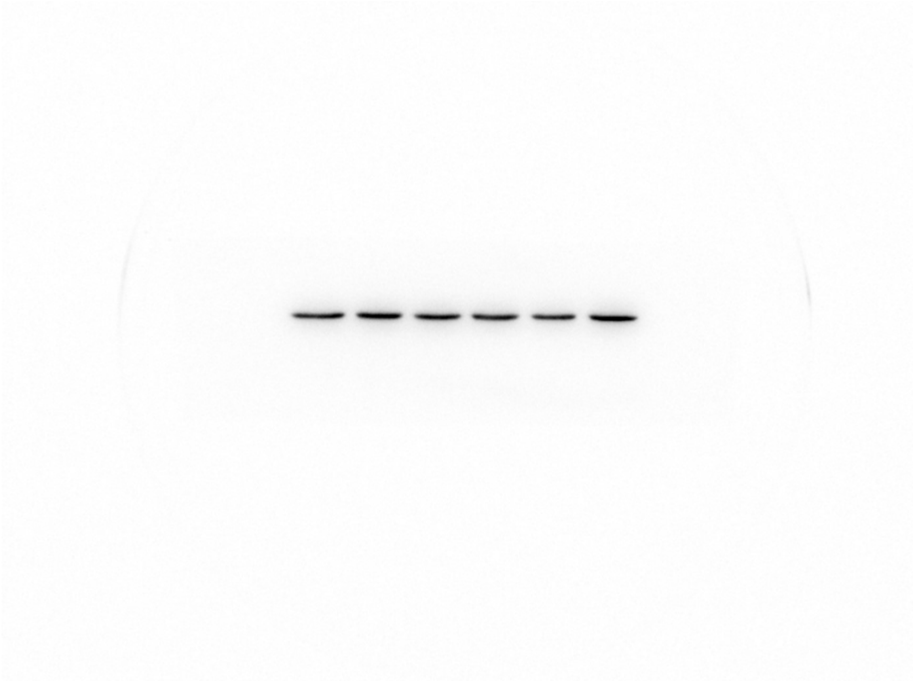

Figure.11. Original western-blot figure of Figure 8 in manuscript. CQAF, β-actin. From left to right: non-treated control group, TNF-α group, CQAF (100 μg/mL) group, CQAF (200 μg/mL) group, CQAF (400 μg/mL) group, quality control sample.

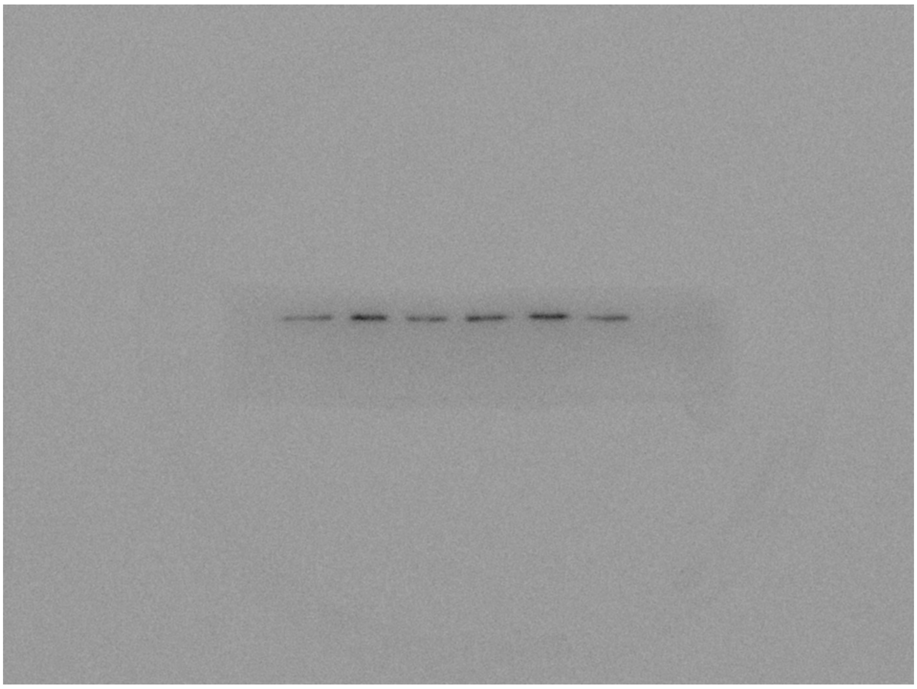

Figure 12. Original western-blot figure of Figure 8 in manuscript. CQAF, IκBα. From left to right: quality control sample, non-treated control group, TNF-α group, CQAF (100 μg/mL) group, CQAF (200 μg/mL) group, CQAF (400 μg/mL) group.

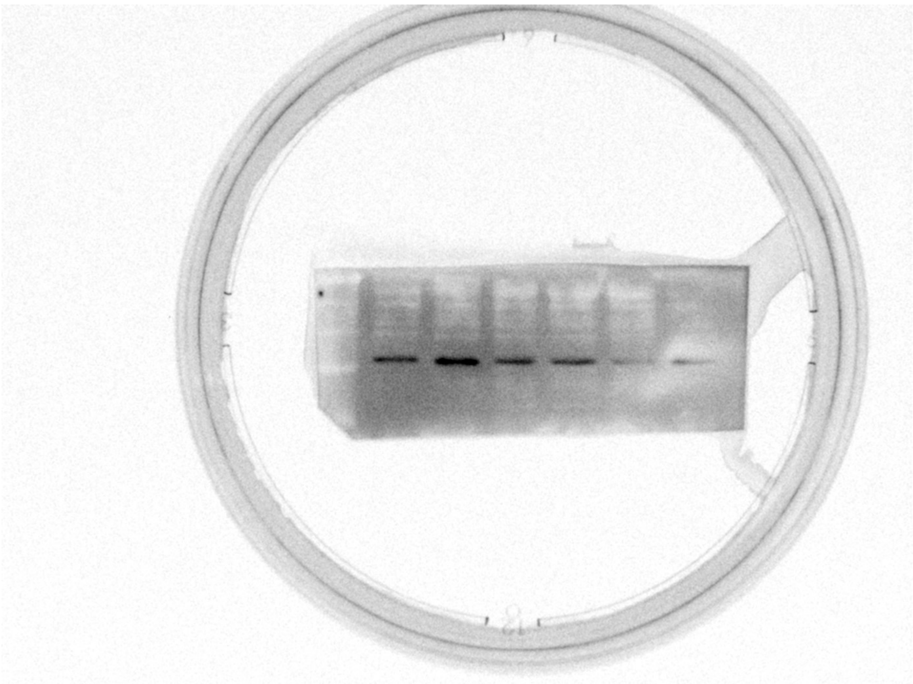

Figure 13. Original western-blot figure of Figure 8 in manuscript. CQAF, p65. From left to right: non-treated control group, TNF-α group, CQAF (100 μg/mL) group, CQAF (200 μg/mL) group, CQAF (400 μg/mL) group, quality control sample.

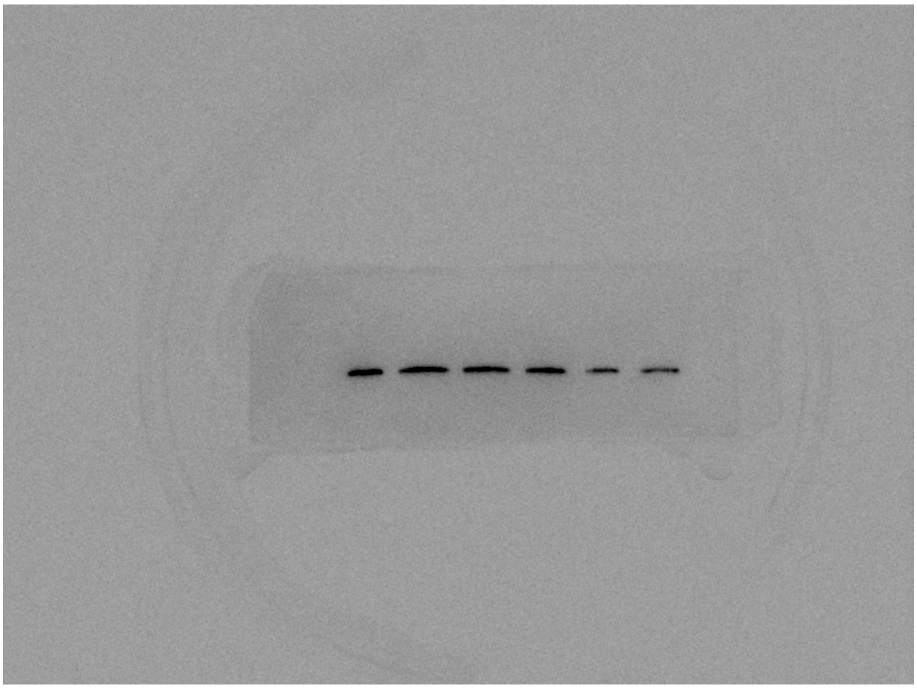

Figure 14 Original western-blot figure of Figure 8 in manuscript. CQAF, p-IκBα. From left to right: non-treated control group, TNF-α group, CQAF (100 μg/mL) group, CQAF (200 μg/mL) group, CQAF (400 μg/mL) group, quality control sample.

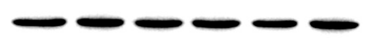

Figure 15. Original western-blot figure of Figure 9 in manuscript. CDLF, β-actin. From left to right: non-treated control group, TNF-α group, CDLF (100 μg/mL) group, CDLF (200 μg/mL) group, CDLF (400 μg/mL) group, quality control sample.

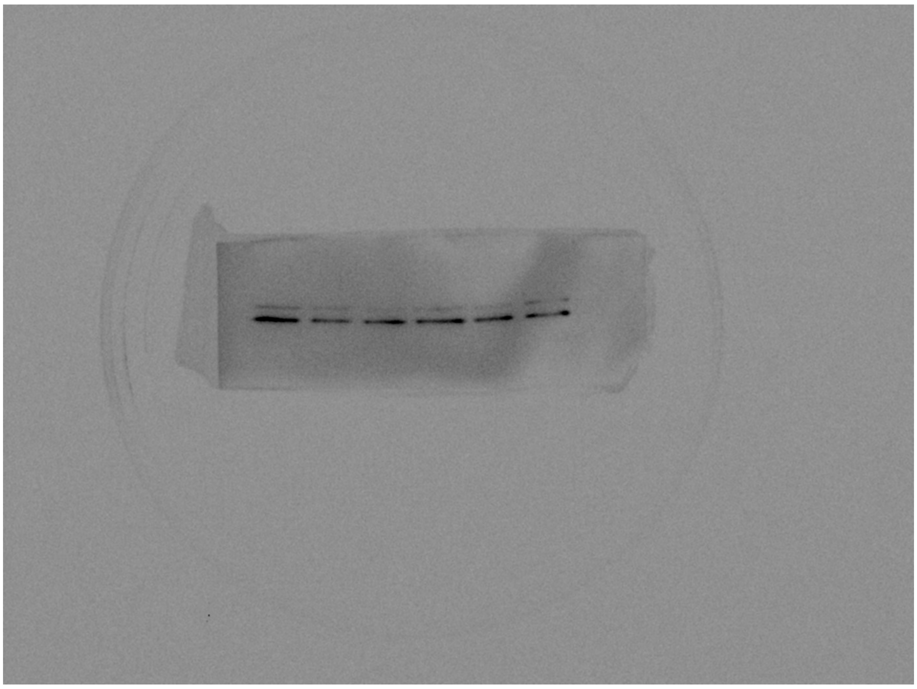

Figure 16. Original western-blot figure of Figure 9 in manuscript. CDLF, $ERK_{1/2}$. From left to right: quality control sample, non-treated control group, TNF-α group, CDLF (100 μg/mL) group, CDLF (200 μg/mL) group, CDLF (400 μg/mL) group.

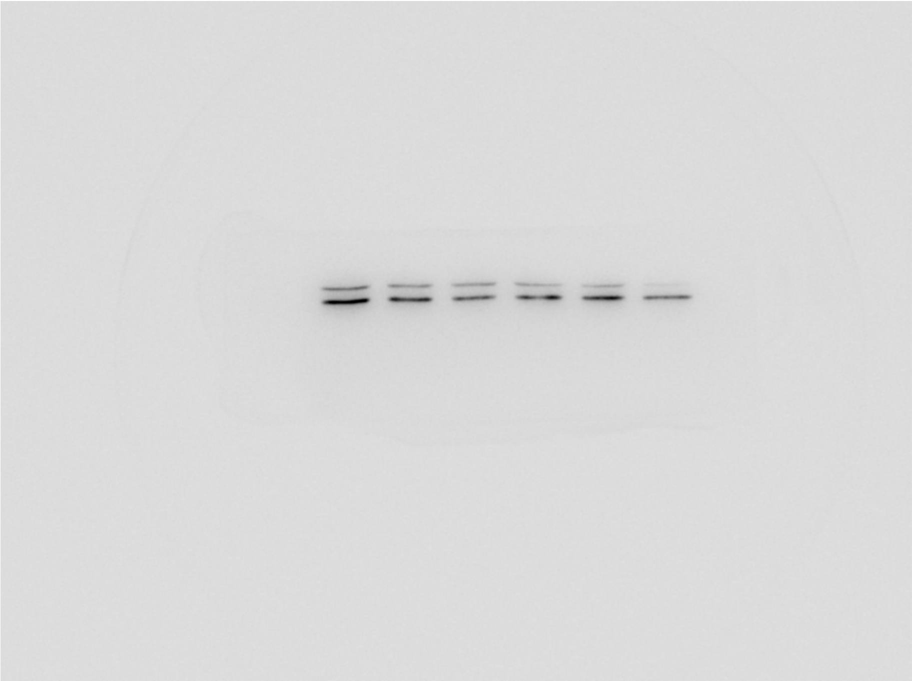

Figure 17. Original western-blot figure of Figure 9 in manuscript. CDLF, JNK. From left to right: quality control sample, non-treated control group, TNF-α group, CDLF (100 μg/mL) group, CDLF (200 μg/mL) group, CDLF (400 μg/mL) group.

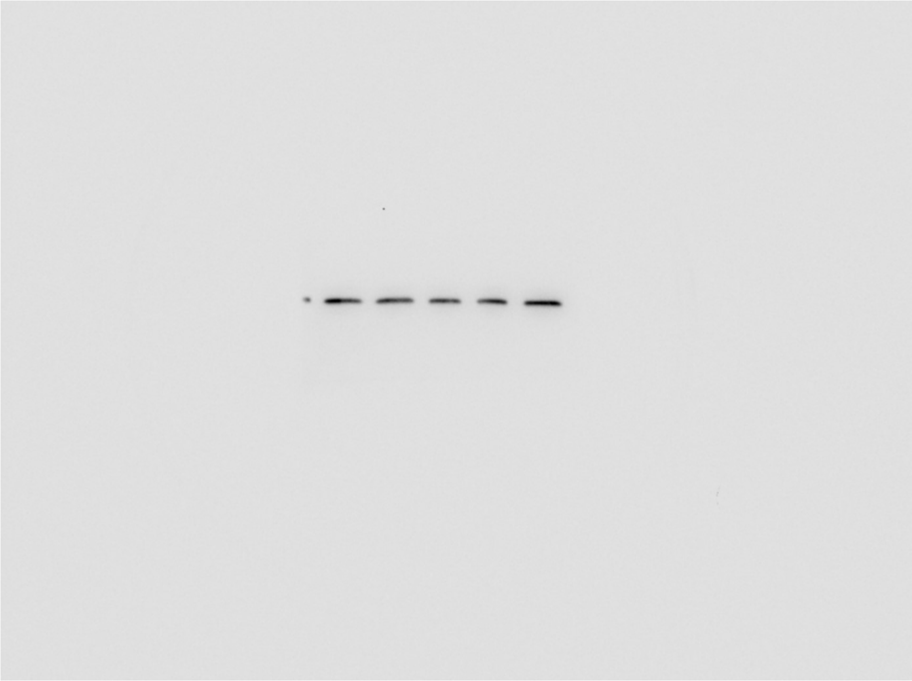

Figure 18. Original western-blot figure of Figure 9 in manuscript. CDLF, p38. From left to right: non-treated control group, TNF-α group, CDLF (100 μg/mL) group, CDLF (200 μg/mL) group, CDLF (400 μg/mL) group.

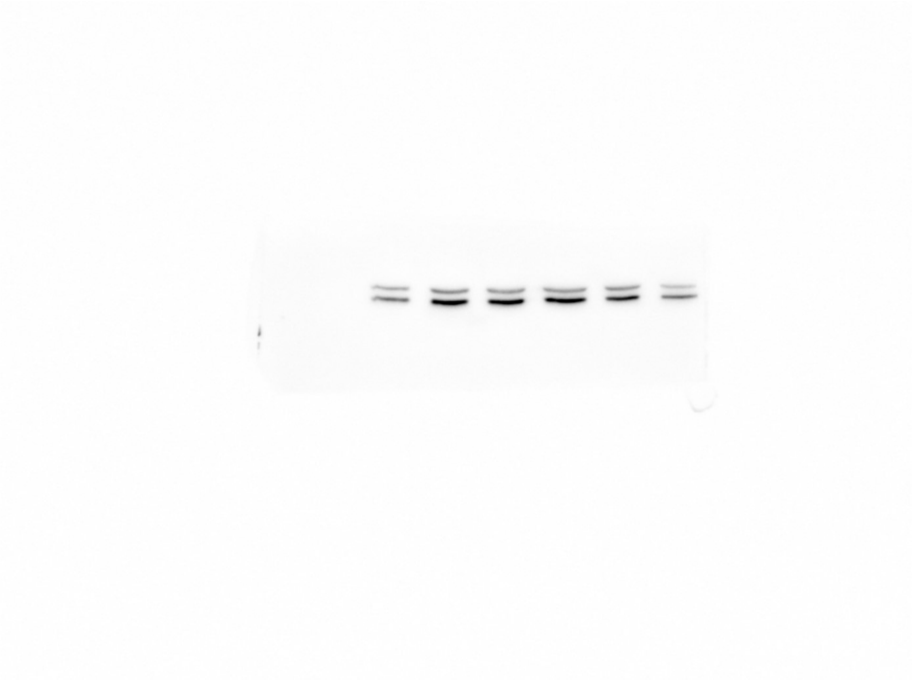

Figure 19 Original western-blot figure of Figure 9 in manuscript. CDLF, p-$ERK_{1/2}$. From left to right: non-treated control group, TNF-α group, CDLF (100 μg/mL) group, CDLF (200 μg/mL) group, CDLF (400 μg/mL) group, quality control sample.

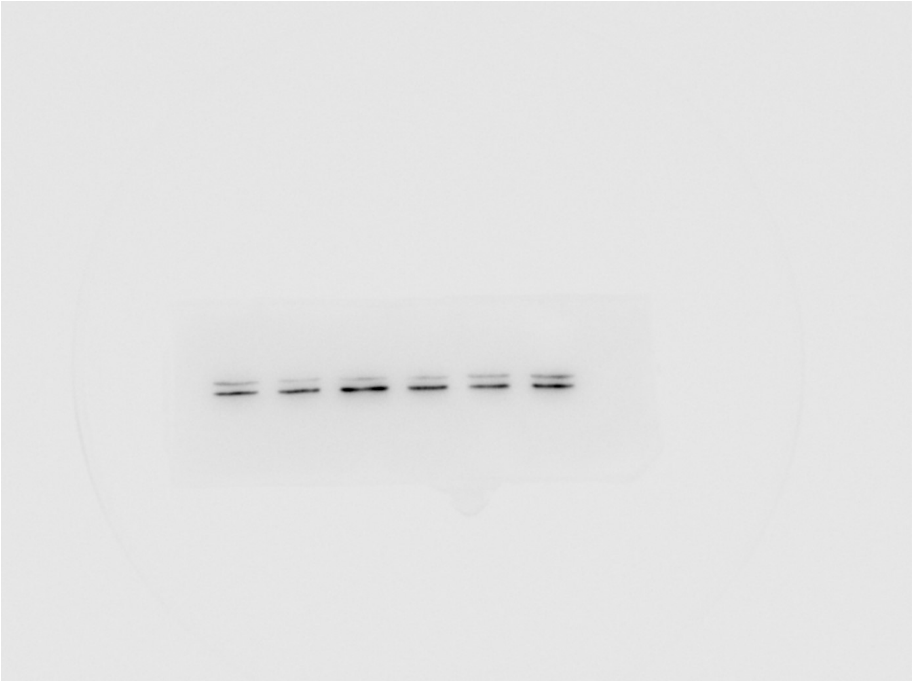

Figure 20. Original western-blot figure of Figure 9 in manuscript. CDLF, p-JNK. From left to right: quality control sample, non-treated control group, TNF-α group, CDLF (100 μg/mL) group, CDLF (200 μg/mL) group, CDLF (400 μg/mL) group.

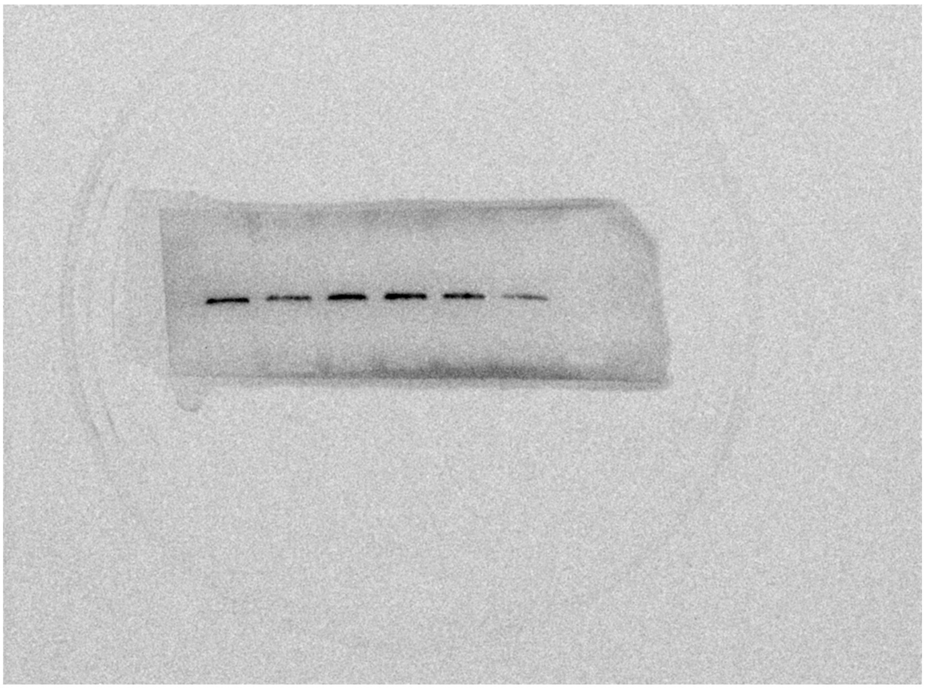

Figure 21. Original western-blot figure of Figure 9 in manuscript. CDLF, p-p38. From left to right: quality control sample, non-treated control group, TNF-*α* group, CDLF (100 μg/mL) group, CDLF (200 μg/mL) group, CDLF (400 μg/mL) group.

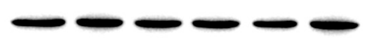

Figure 22. Original western-blot figure of Figure 9 in manuscript. CQAF, β-actin. From left to right: non-treated control group, TNF-*α* group, CQAF (100 μg/mL) group, CQAF (200 μg/mL) group, CQAF (400 μg/mL) group, quality control sample

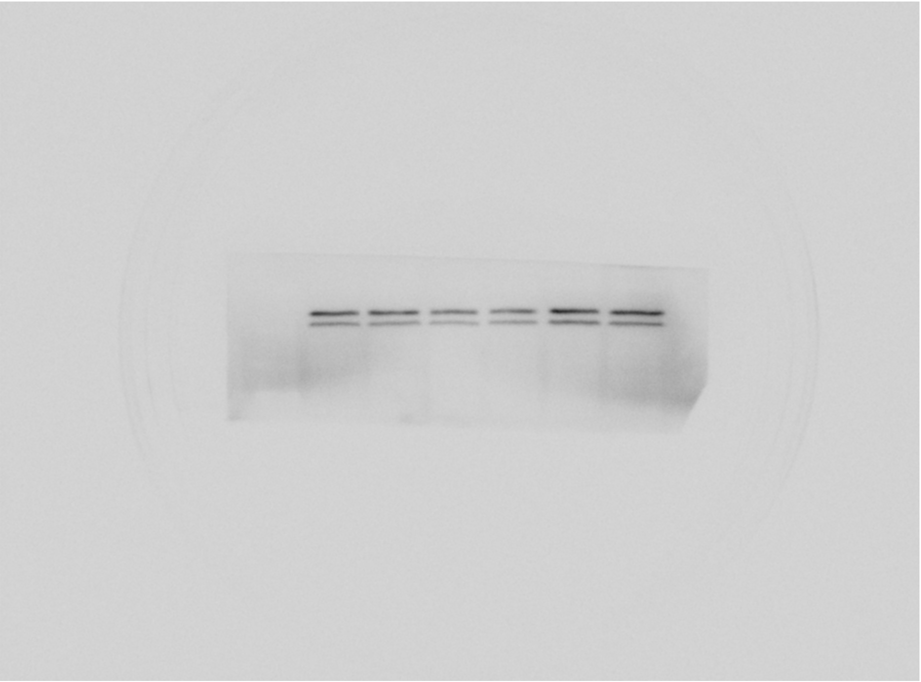

Figure 23. Original western-blot figure of Figure 9 in manuscript. CQAF, $ERK_{1/2}$. From left to right: quality control sample, non-treated control group, TNF-α group, CQAF (100 μg/mL) group, CQAF (200 μg/mL) group, CQAF (400 μg/mL) group.

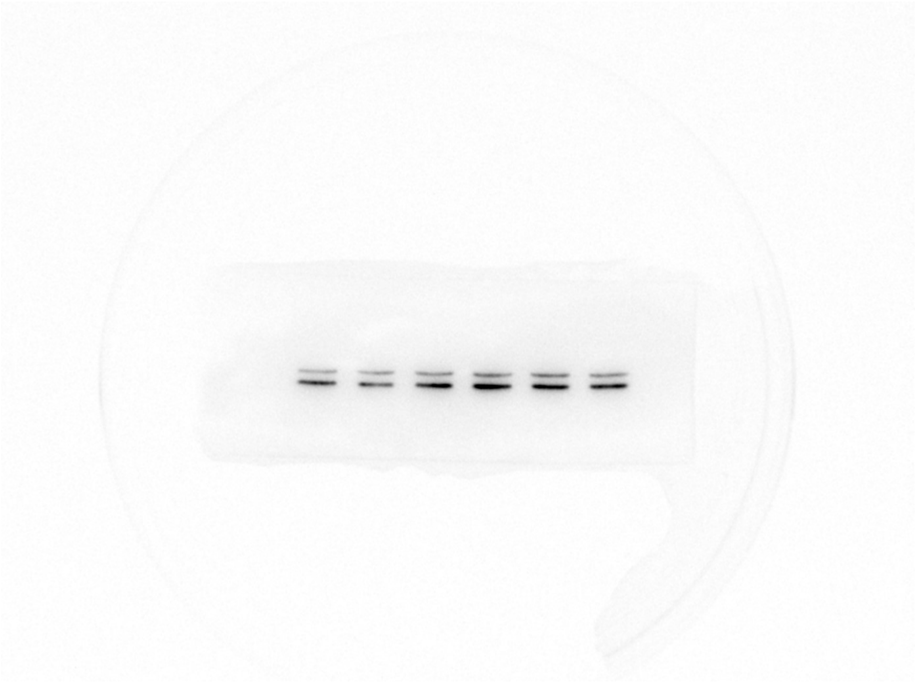

Figure 24. Original western-blot figure of Figure 9 in manuscript. CQAF, JNK. From left to right: non-treated control group, TNF-α group, CQAF (100 μg/mL) group, CQAF (200 μg/mL) group, CQAF (400 μg/mL) group, quality control sample.

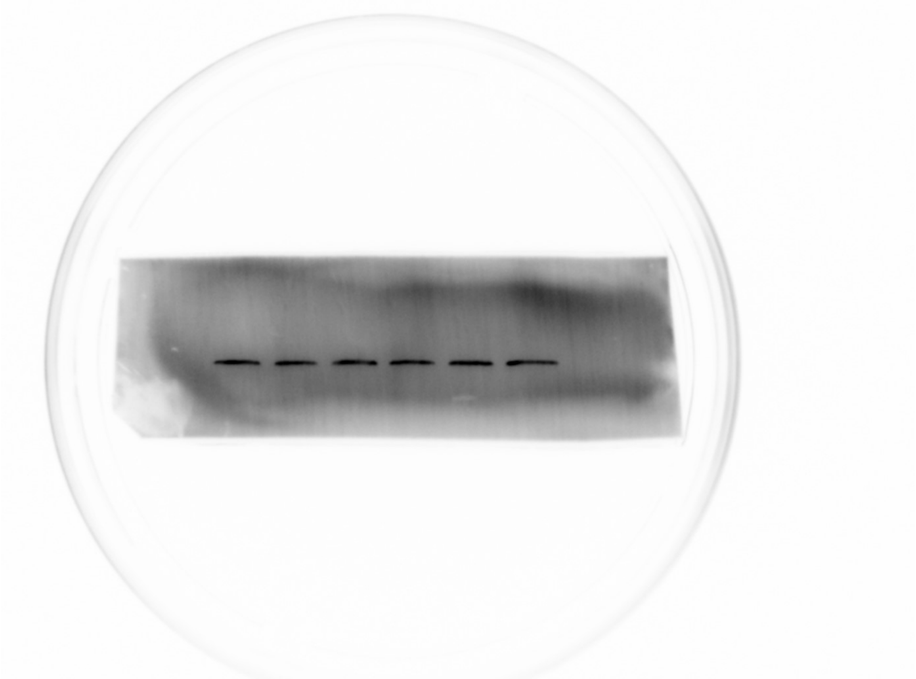

Figure 25. Original western-blot figure of Figure 9 in manuscript. CQAF, p38. From left to right: non-treated control group, TNF-α group, CQAF (100 μg/mL) group, CQAF (200 μg/mL) group, CQAF (400 μg/mL) group, quality control sample.

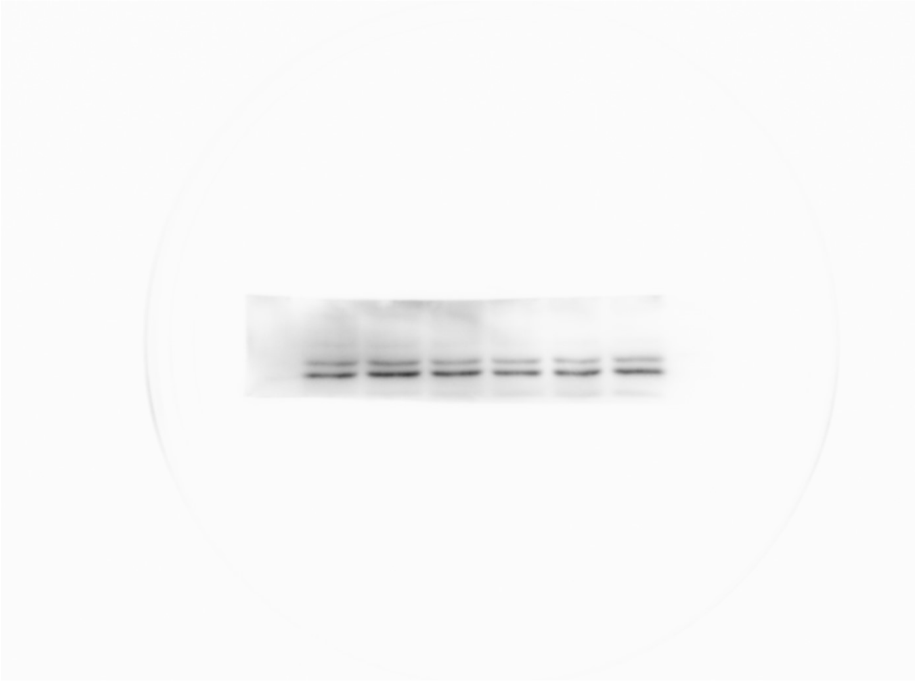

Figure 26. Original western-blot figure of Figure 9 in manuscript. CQAF, p-$ERK_{1/2}$. From left to right: non-treated control group, TNF-α group, CQAF (100 μg/mL) group, CQAF (200 μg/mL) group, CQAF (400 μg/mL) group, quality control sample.

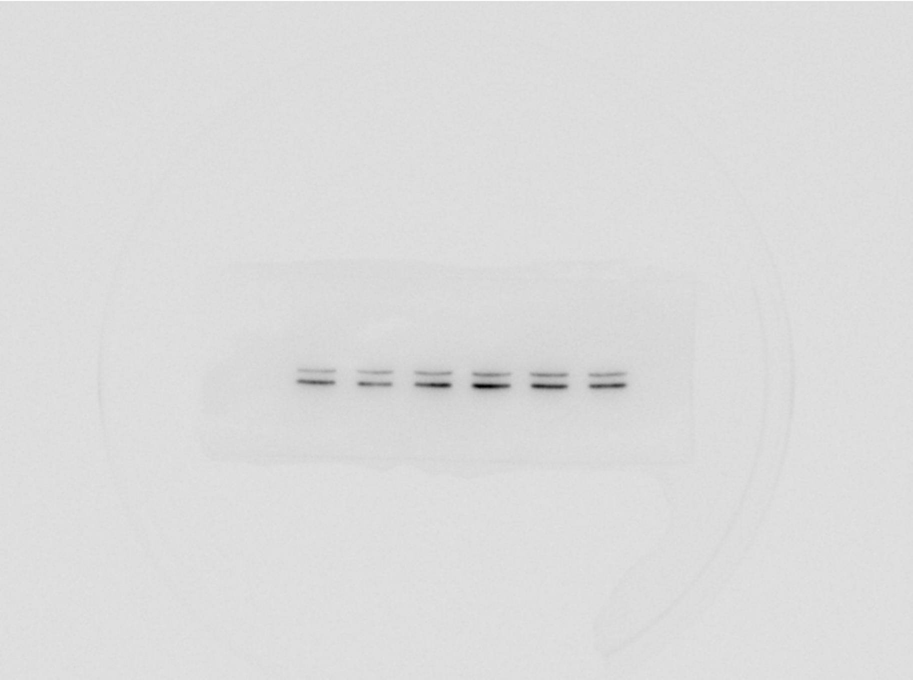

Figure 27. Original western-blot figure of Figure 9 in manuscript. CQAF, p-JNK. From left to right: quality control sampl, non-treated control group, TNF-α group, CQAF (100 μg/mL) group, CQAF (200 μg/mL) group, CQAF (400 μg/mL) group.

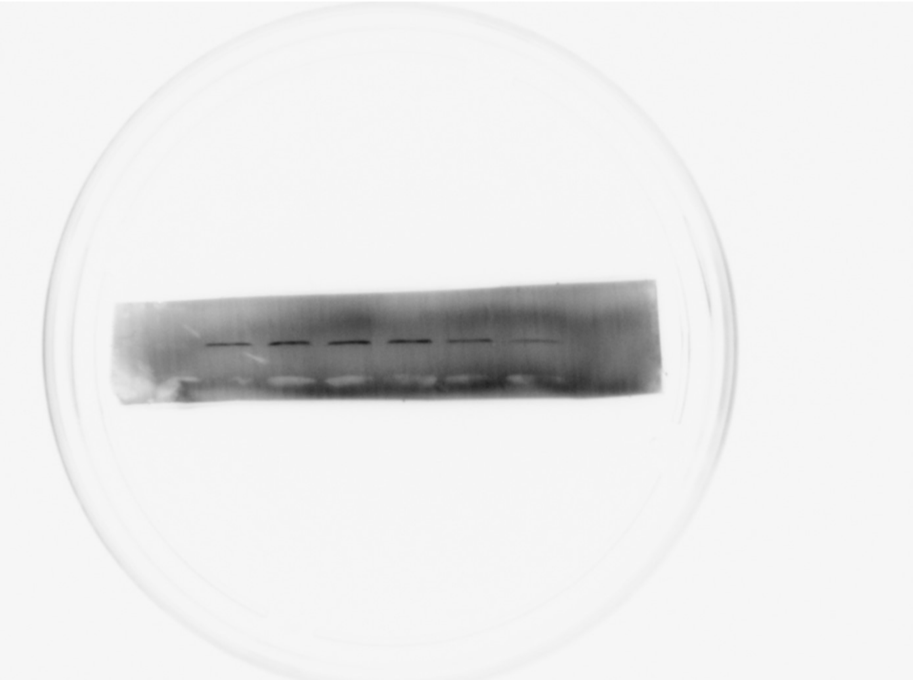

Figure 28. Original western-blot figure of Figure 9 in manuscript. CQAF, p-p38. From left to right: non-treated control group, TNF-α group, CQAF (100 μg/mL) group, CQAF (200 μg/mL) group, CQAF (400 μg/mL) group, quality control sample.

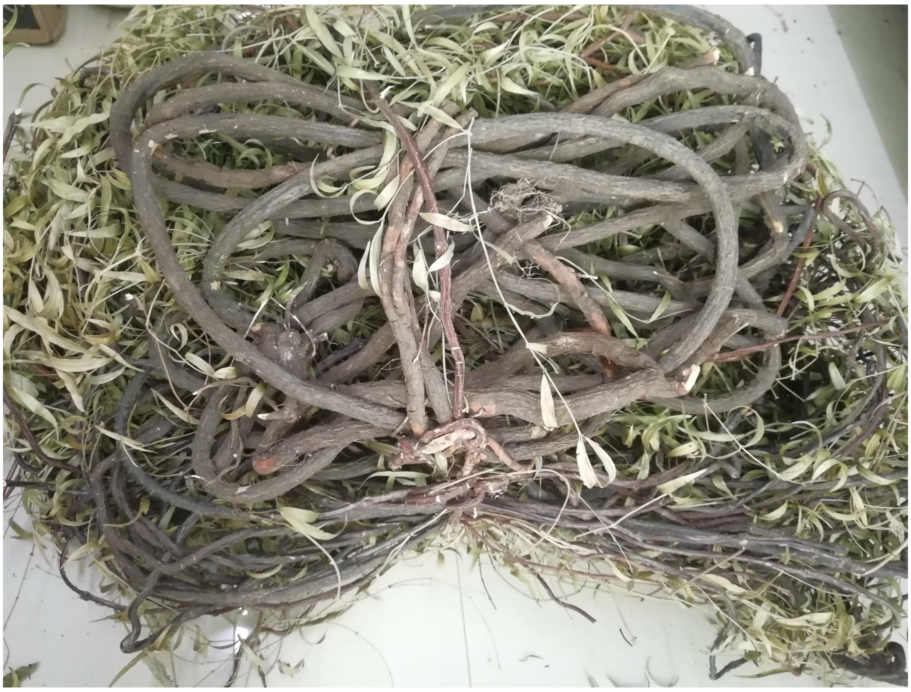

Figure 29. Whole plant of *Periploca forrestii* used in the present study.

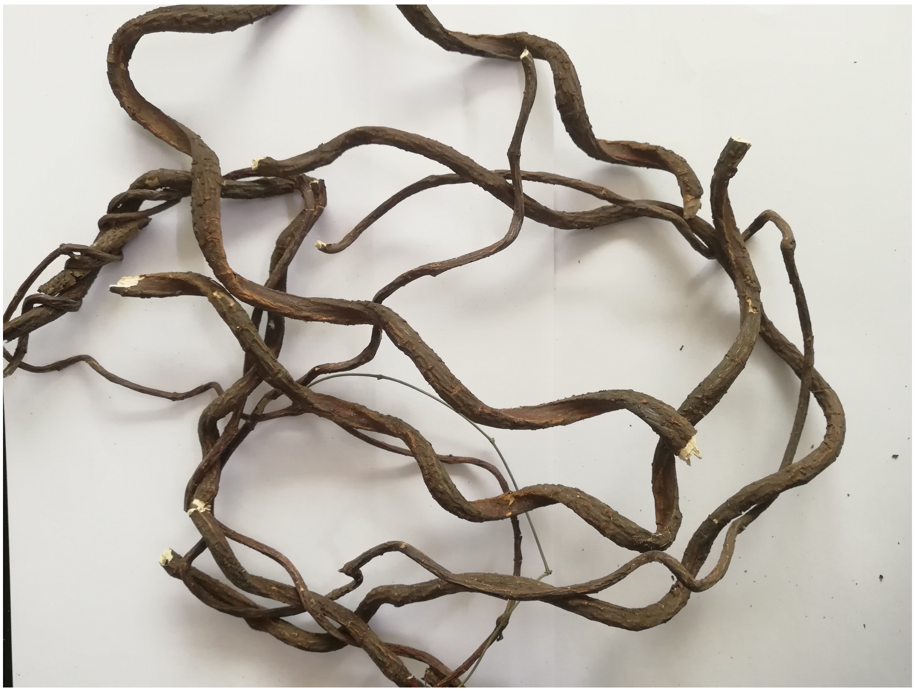

Figure 30. Stalks of *Periploca forrestii* used in the present study.

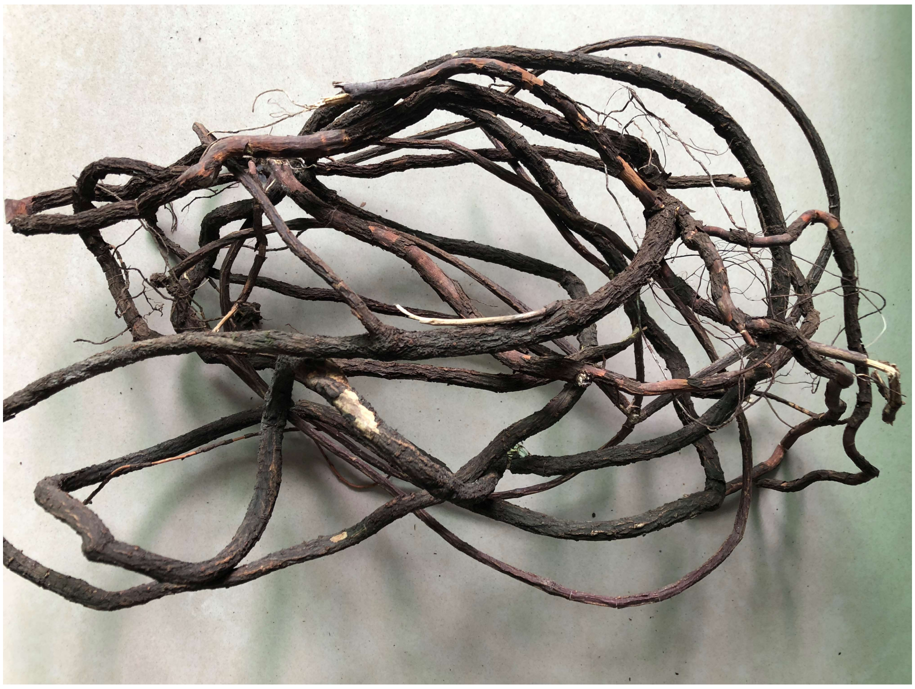

Figure 31. Roots and stalks of *Periploca forrestii* used in the present study.

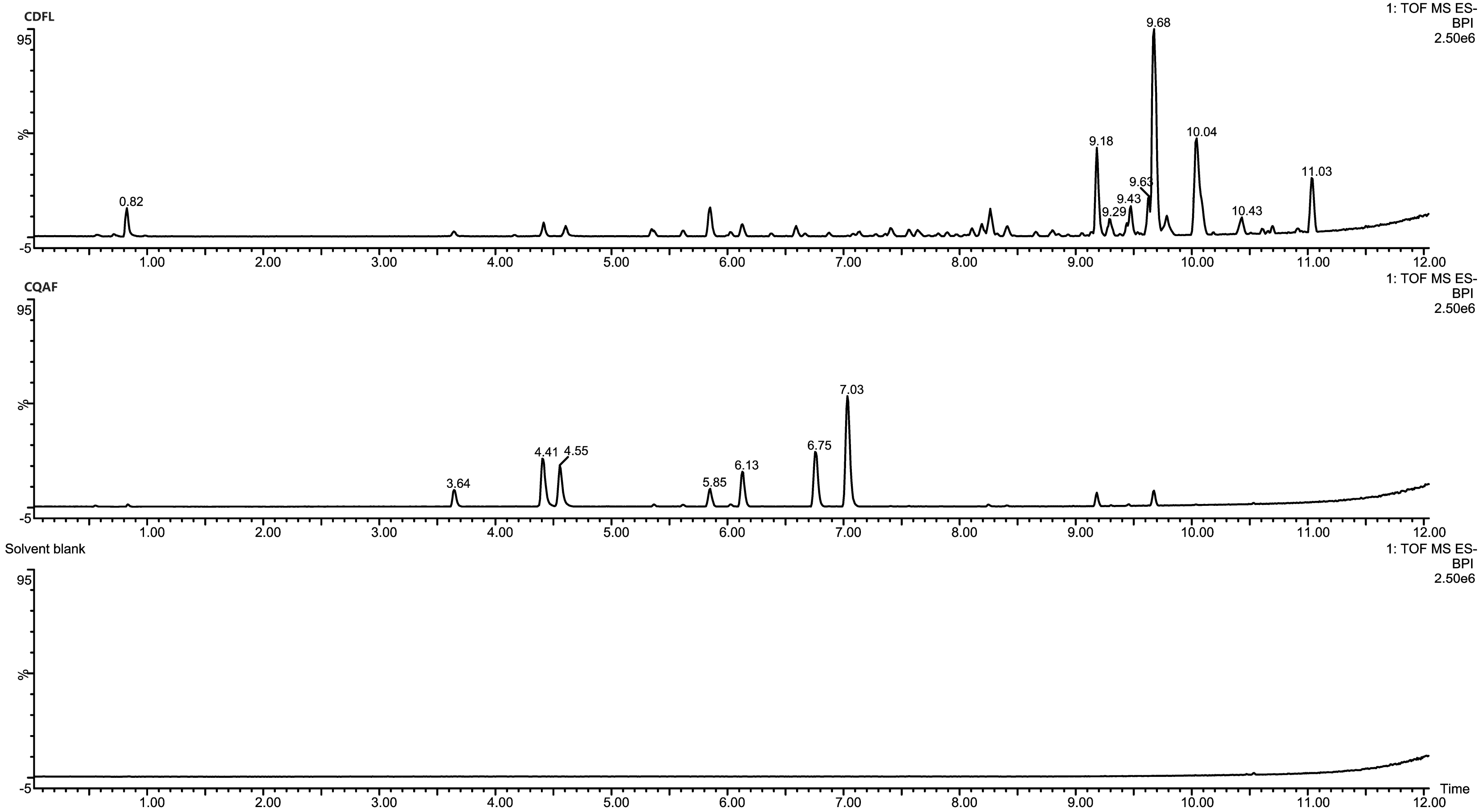

Figure 32. UPLC-QTOF-MS chromatograms of CDFL (0.5 mg/mL) and CQAF (0.05 mg/mL). Negative ion mode.

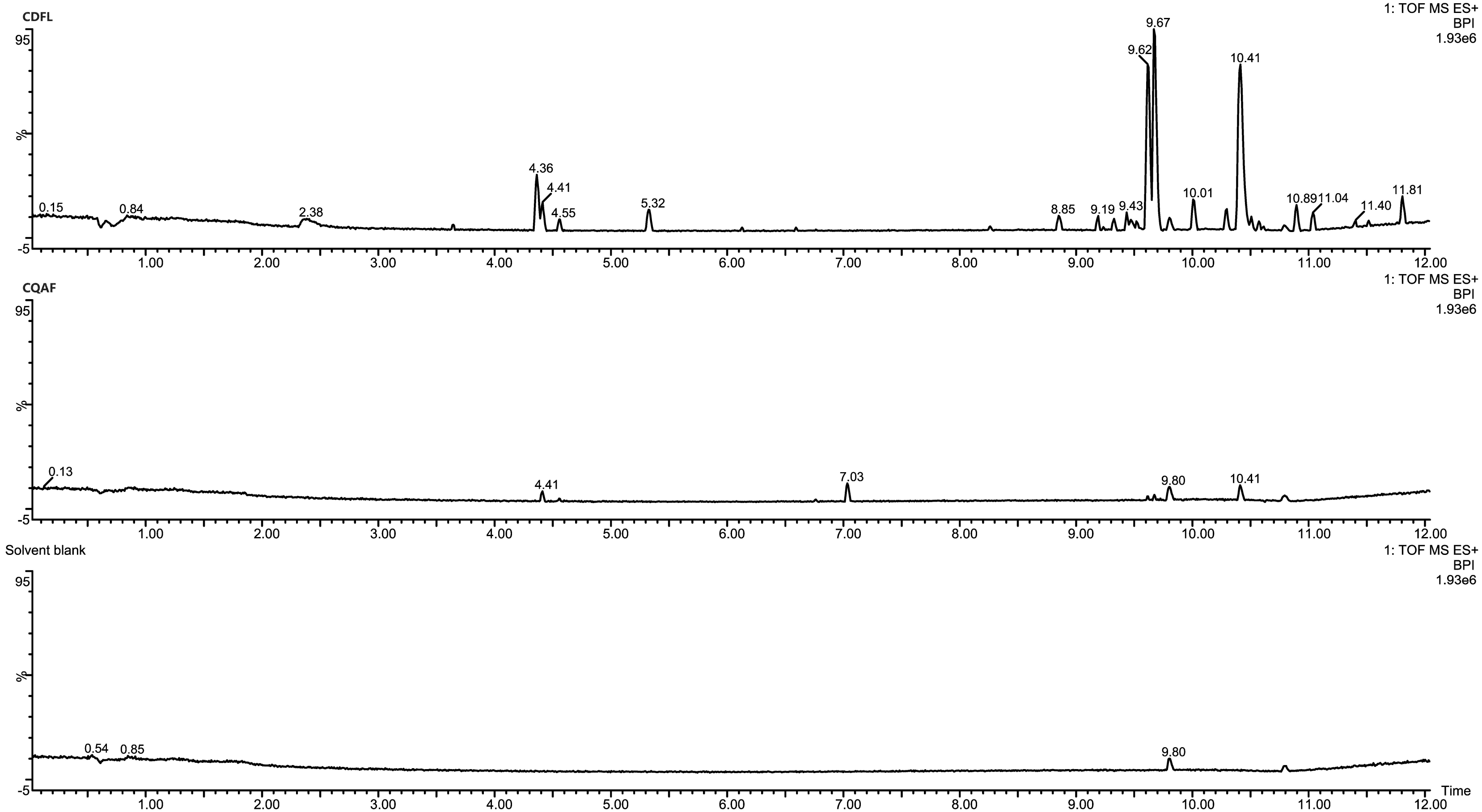

Figure 33. UPLC-QTOF-MS chromatograms of CDFL (0.5 mg/mL) and CQAF (0.05 mg/mL). Positive ion mode.
